# Organoids: development and applications in disease models, drug discovery, precision medicine, and regenerative medicine

**DOI:** 10.1002/mco2.735

**Published:** 2024-09-21

**Authors:** Qigu Yao, Sheng Cheng, Qiaoling Pan, Jiong Yu, Guoqiang Cao, Lanjuan Li, Hongcui Cao

**Affiliations:** ^1^ State Key Laboratory for the Diagnosis and Treatment of Infectious Diseases National Clinical Research Center for Infectious Diseases Collaborative Innovation Center for Diagnosis and Treatment of Infectious Diseases National Medical Center for Infectious Diseases The First Affiliated Hospital Zhejiang University School of Medicine Hangzhou China; ^2^ Zhejiang Key Laboratory for Diagnosis and Treatment of Physic‐Chemical and Aging‐Related Injuries Hangzhou China

**Keywords:** animal models, disease model, drug screening organoids, personalized medicine

## Abstract

Organoids are miniature, highly accurate representations of organs that capture the structure and unique functions of specific organs. Although the field of organoids has experienced exponential growth, driven by advances in artificial intelligence, gene editing, and bioinstrumentation, a comprehensive and accurate overview of organoid applications remains necessary. This review offers a detailed exploration of the historical origins and characteristics of various organoid types, their applications—including disease modeling, drug toxicity and efficacy assessments, precision medicine, and regenerative medicine—as well as the current challenges and future directions of organoid research. Organoids have proven instrumental in elucidating genetic cell fate in hereditary diseases, infectious diseases, metabolic disorders, and malignancies, as well as in the study of processes such as embryonic development, molecular mechanisms, and host–microbe interactions. Furthermore, the integration of organoid technology with artificial intelligence and microfluidics has significantly advanced large‐scale, rapid, and cost‐effective drug toxicity and efficacy assessments, thereby propelling progress in precision medicine. Finally, with the advent of high‐performance materials, three‐dimensional printing technology, and gene editing, organoids are also gaining prominence in the field of regenerative medicine. Our insights and predictions aim to provide valuable guidance to current researchers and to support the continued advancement of this rapidly developing field.

## Graphical abstract

Four main applications of organoids. The main applications of organoids include the construction of disease models, drug screening and toxicity evaluation, precision medicine, and regenerative medicine.

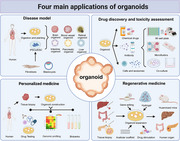

## 1 INTRODUCTION

In 1998 and 2006, the application of human embryonic stem cells (hESCs) and induced pluripotent stem cells (iPSCs) led to significant advancements in the understanding of mechanisms underlying stem cell fate determination.^1^, ^2^ In 2009, Clevers et al.^3^ first constructed intestinal organoids, sparking a surge of interest in organoid research. Organoid systems can encapsulate numerous essential characteristics of stem cell niches and the tissues they generate, replicating the structure, cellular composition, and self‐renewal dynamics of the original tissues.^4^ Thus far, researchers have developed retinal, brain, kidney, intestinal, and gastric organoids, using iPSCs or adult stem cells (ASCs).^5^, ^6^, ^7^, ^8^, ^9^

Organoid technology serves as a critical bridge between conventional cell lines and in vivo models. Organoids are widely utilized for studies of disease models, drug discovery, precision medicine, and regenerative medicine.^10^, ^11^ However, conventional organoid cultures often lack surrounding stromal cells, immune cells, and vascular endothelial cells. Additionally, variations in induction times and methods for iPSCs, as well as batch inconsistencies in Matrigel, constitute important challenges. Issues related to the maturity and reproducibility of organoids limit their application and development, particularly in disease modeling and regenerative medicine.

In recent years, the integration of artificial intelligence (AI) with high‐performance materials and instruments has created new opportunities for organoid applications, offering groundbreaking insights into human physiology and pathology.^12^, ^13^ When combined with AI technology, microfluidics, and imaging analysis, organoids facilitate rapid functional drug testing and precision medical diagnostics for patients.^14^, ^15^ Additionally, advancements in hydrogels, biological scaffolds, in vitro vascularization, and tissue engineering have greatly enhanced the role of organoids in regenerative medicine.^16^ For instance, human brain organoids have been successfully transplanted into the striatum of NOD/SCID/γ‐chain knockout mice, human bile duct organoids have been implanted into human liver tissue, and human intestinal organoids have been used in clinical trials for ulcerative colitis.^17^, ^18^, ^19^

In summary, an understanding of the latest applications and advancements in organoids is crucial, particularly considering their integration with bioengineering, AI technology, and organoid innovation. This review highlights the characteristics of various organoids, engineering methods, and the most recent applications of organoid technology.

We first introduce the characteristics of organoid construction methods and explore how bioengineering approaches can enhance their utility in research and treatment. We then discuss the applications of organoid technology in disease modeling, drug screening, precision therapy, and regenerative medicine. Finally, we highlight the current limitations and future prospects of organoid development, aiming to further enhance their effectiveness in both research and therapy.

## 2 DEVELOPMENTS AND ADVANCES IN ORGANOIDS

**FIGURE 1:**
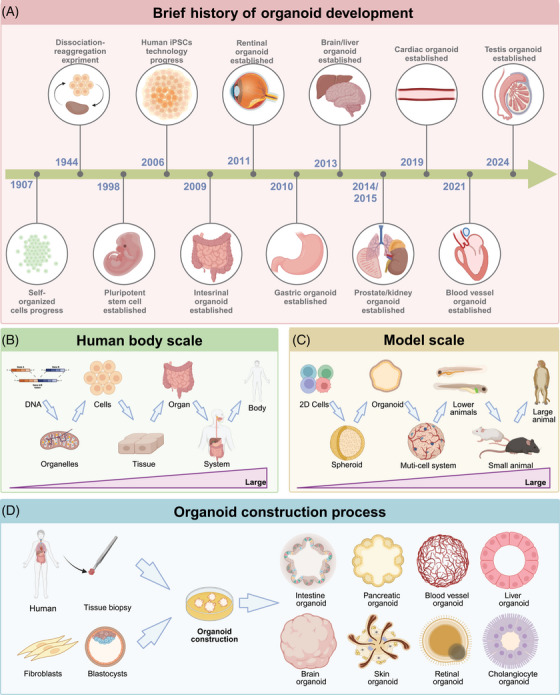
Organoid construction process. (A) Chronological history of organoid establishment for each organ. From left to right in chronological order. (B) Organisms include system hierarchies from the DNA level to the entire body. (C) Model scale from cell lines to large animals such as monkeys. (D) Strategies for forming organoids in vitro. Embryonic stem cells (ESC), induced pluripotent stem cells (iPSC), and adult stem cells (AdSC) generate liver, intestine, retinal, blood vessel, brain, skin, cholangiocyte, kidney, and other organoids.

The self‐organizing properties of cells are generally traced back to 1907, when H.V. Wilson demonstrated that sponge cells could self‐organize to regenerate an entire organism.^20^ In 1998 and 2006, the use of hESCs and iPSCs sparked a wave of research into the mechanisms of stem cell fate determination.^1^, ^2^ In 2009, Clevers et al.^3^ demonstrated that the provision of leucine‐rich repeat‐containing G‐protein coupled receptor 5 (Lgr5) stem cells or isolated crypts with an appropriate niche—consisting of Matrigel, epidermal growth factor, Wingless‐related integration site (WNT), Noggin, R‐spondin‐1, and other cytokines—could lead to the formation of three‐dimensional (3D) intestinal organoids. The use of ASC technology to construct organoids laid the foundation for subsequent developments. Between 2009 and 2024, organoids of the retina, prostate, brain, liver, kidney, heart, blood vessels, and more have emerged.^21^, ^22^, ^23^, ^24^, ^25^ These organoids encapsulate the genotypes, phenotypes, and cellular functions of their respective organs, achieving significant breakthroughs in biological modeling (Figure 1A).^26^

From a biomedical perspective, two‐dimensional (2D) cell cultures, organoids, multicellular systems, and models ranging from lower animals to higher mammals each summarize bodily functions and processes, spanning from the molecular level to cells, tissues, organs, and whole organisms.^11^, ^27^, ^28^ Organoids encapsulate the genetic profiles, cellular characteristics, cell–cell interactions, cell–matrix interactions, and physiological functions of organ‐specific cells. Their unique features and suitability for scalable culture bridge gaps in existing model systems, thus enhancing our understanding of human development and disease (Figure 1B,C).

Organoids are generated from hESCs, iPSCs, and ASCs, simulating physiological tissue development.^29^ iPSCs are produced by reprogramming somatic cells, whereas hESCs are derived from the inner cell mass of blastocysts and induced to differentiate into specific lineages enabling the formation of organ‐specific organoids, commonly observed in the brain, kidney, heart, and retina.^23^, ^30^, ^31^, ^32^ The cell composition of organoids derived from hESCs and iPSCs is relatively complex, often including mesenchymal, epithelial, and even endothelial components; however, their development is often time consuming. Additionally, specific ASCs can be expanded in vitro under defined culture conditions (such as epidermal growth factor, fibroblast growth factor, Noggin, and WNT pathway activation) to control self‐renewal and differentiation, resulting in the self‐organizing organoids frequently found in the intestine, liver, pancreas, and various cancers (Figure 1D).^33^, ^34^, ^35^, ^36^ ASC‐derived organoids are closer in maturity to adult tissues, making them more suitable for the modeling of adult tissue repair and viral infections. In summary, evidence suggests that organoids derived from hESCs, iPSCs, and ASCs can serve as complementary tools in future scientific research and potential personalized medicine. Here, we discuss the development and limitations of organoids according to their germ layer origin (ectoderm: brain, retina; mesoderm: kidney, heart; endoderm: lung, liver, and intestine). Finally, we explore engineering approaches to enhance organoid maturity, reproducibility, and assessment methods.

### 2.1 Advancements and limitations of brain organoids

The human brain comprises a diverse array of cell types and presents complex challenges, including intricate neural circuits, vascular circulation, and immune evasion.^37^ Animal models and 2D cell cultures often fail to fully address these issues due to ethical concerns and limitations in model complexity.^38^ However, brain organoids replicate several crucial aspects of early human brain development, encompassing molecular, cellular, structural, and functional dimensions.^26^ In 2013, Lancaster et al.^24^ pioneered the use of iPSC technology to construct brain organoids that exhibited key characteristics of human cortical development, including a distinct progenitor zone rich in radial glial stem cells. In 2018, Mansour et al.^39^ successfully transplanted human brain organoids into mouse brains, enabling vascularization and functional integration. In 2020, Pellegrini et al.^40^ advanced this work by creating human choroid plexus organoids with selective barriers and cerebrospinal fluid‐like secretion in independent compartments. Most recently, in 2023, Schafer et al.^41^ constructed vascularized brain organoids containing microglia, effectively modeling the ongoing neuroinflammatory processes observed in the brains of autistic children.

Brain organoids have successfully recapitulated central nervous system viral infections and genetic brain diseases. For example, there is evidence that Zika virus infection causes a reduction in brain organoid size and a loss of surface folds.^42^ Severe acute respiratory syndrome coronavirus 2 (SARS‐CoV‐2) infection in human brain organoids leads to neuron‐neuron and neuron‐glial cell fusion, resulting in cell death and synaptic loss.^43^, ^44^ In 2019, Velasco et al.^45^ utilized four distinct protocols to produce 3D brain organoids, revealing that early brain region patterning initiates cell specialization and maturation; extrinsic factors also play crucial roles.^45^ Additionally, serum‐treated brain organoids recapitulate Alzheimer's disease‐like pathology, inducing levels of β‐secretase 1 and glycogen synthase kinase 3 α/β that lead to increased levels of Aβ and p‐Tau.^46^

Despite the extensive insights provided by brain organoids, future developments should address several key considerations. First, brain organoids offer a unique opportunity for scientific exploration of consciousness, underscoring the importance of further ethical research to assess potential risks associated with human brain organoids and human–animal chimeric organoids.^47^, ^48^ Second, although Trujillo et al.^49^ found that cortical organoids can generate continuous electrical activity signals, questions remain concerning whether neurons in brain organoids can establish proper synaptic connections to form mature cortical circuits. Third, iPSC‐derived brain organoids require further maturation, particularly through the inclusion of essential components (e.g., vascular endothelium, microglia, and the blood–brain barrier) that are critical for the creation of a fully functional brain model.^50^

### 2.2 Advancements and limitations of retinal organoids

The retina is a critical structure in the eye responsible for processing photoreceptive information. The differentiation of retinal organoids mirrors the formation of optic vesicles and can include most retinal neuronal cell types, such as cone cells, ganglion cells, bipolar cells, horizontal cells, and amacrine cells.^51^ In 2011, Eiraku et al.^52^ reported the dynamic and autonomous formation of optic cups (retinal primordia) from 3D cultures of mouse embryonic stem cell aggregates. Nakano et al.^53^ later discovered that optic cup structures could self‐organize in hESC cultures; the neural retina formed a thick layer that spontaneously curved into an apical structure. In 2014, Zhong et al.^54^ achieved advanced maturation of photoreceptors in hiPSC‐derived retinal organoids, demonstrating the formation of outer segment disks and light sensitivity. In 2019, Achberger et al.^55^ combined organoid and organ‐on‐chip technologies to create a sophisticated human multilayered tissue retinal platform. This model provided vessel‐like perfusion; for the first time, it captured the interactions between in vitro matured photoreceptors and retinal pigment epithelium.

Retinal organoids are a valuable tool for studies of eye development and visual function, as well as the acquisition of retinal tissue. However, they continue to exhibit deficiencies that require improvement. For example, although mature retinal organoids develop photoreceptor outer segment‐like structures containing opsins, they do not exhibit proper disk stacking and orientation.^56^ Additionally, retinal organoids lack vasculature and immune‐related cells; they are unable to replicate the foveal structure needed to study diseases such as macular degeneration.^57^, ^58^

### 2.3 Advancements and limitations of kidney organoids

Kidney development is believed to originate from the interaction between two embryonic cell populations: ureteric bud and metanephric mesenchyme.^59^ In 2014, Taguchi et al.^60^ and Takasato et al.^60^, ^61^ successfully differentiated human iPSCs and hESCs into metanephric mesenchyme organoids and organoids containing nephron structures. Combes et al.^62^ utilized single‐cell sequencing to compare kidney organoids with human fetal kidneys, revealing a high fidelity in nephron, stromal, and endothelial cell types. Relative to immortalized human podocyte lines, podocytes in kidney organoids demonstrated correct basolateral polarity and gene expression profiles, offering a more accurate representation of in vivo conditions.^63^, ^64^ Considering that the kidney is the primary organ responsible for urine concentration, drug metabolism, and toxin filtration, human kidney organoids have been used to simulate autophagy processes in tacrolimus‐damaged renal cells, thereby revealing the mechanisms of drug‐induced nephrotoxicity.^51^ Concerning pharmacological screening platforms that use organoids, Lawlor et al.^65^ in 2021 employed 3D bioprinting technology to precisely manipulate the biophysical properties of kidney organoids and assess the relative toxicities of drugs such as aminoglycosides.

Renal organoid research must address several key points as it progresses toward the creation of a fully functional in vitro kidney model.^66^ For instance, iPSC‐derived kidney organoids face challenges in fully replicating the characteristics of nongenetic kidney diseases due to the removal of epigenetic marks during reprogramming.^67^ Additionally, although iPSC‐derived kidney organoids contain nephron structures, they exhibit limited functionality, growth, and lifespan, likely due to the absence of blood supply, immune cells, and neural cells.^68^ Furthermore, the complexity of culturing iPSC‐derived kidney organoids and tubules poses challenges in maintaining experimental reproducibility. The excessive use of growth factors can also influence immune cell differentiation. For example, A8301 affects T helper cell polarization by altering transforming growth factor‐β signaling,^69^ and the inclusion of interleukin‐2 in T cell cultures impacts podocyte injury and apoptosis.^70^

### 2.4 Advancements and limitations of cardiac organoids

The heart is the first functional organ to develop during human embryogenesis, consisting of cardiomyocytes, cardiac fibroblasts, and endothelial cells.^71^ The unique 3D morphology of cardiac organoids has significantly advanced the field by enabling studies of cavity formation, wall thickness, ejection fraction during contraction, and endothelial tubulogenesis.^72^ Compared with organoids from other organs, cardiac organoids initially exhibited slower development. In 2017, Giacomelli et al.^73^ demonstrated that hiPSCs could codifferentiate into 3D cardiac microtissues composed of cardiomyocytes and endothelial cells. In 2021, Hofbauer et al.^74^ utilized hiPSC technology to create the first self‐organizing cardiac organoids by regulating the mesodermal WNT–bone morphogenetic protein signaling axis, resulting in chamber‐like cardioids with cavities that replicate cardiac lineage structures. In the same year, Lewis‐Israeli et al.^75^ developed complex luminal structures with multiple cardiac cell types that could reproduce heart field formation and atrioventricular features, reflecting the metabolic disruptions associated with congenital heart defects. Drakhlis et al.^31^ reviewed early heart and foregut development, contributing to the investigation of cardiac genetic defects in vitro. Additionally, cardiac organoids have shown promise in transplantation studies. Between 2018 and 2021, several studies demonstrated the transplantation of hESC‐ and hiPSC‐derived cardiac organoids into mouse brain and abdominal cavities.^76^ Overall, cardiac organoids have rapidly progressed in developmental biology, disease modeling, and transplantation research, gaining substantial attention.

Despite these advancements, cardiac organoids require extensive improvements. For instance, iPSC‐derived organoids continue to exhibit embryonic characteristics.^77^ Currently, they can replicate cell types such as ventricular cardiomyocytes, endothelial cells, and pericytes, but they lack the ability to model the cardiac conduction system that is necessary for studies of arrhythmogenic cardiomyopathy. Additionally, they do not yet incorporate the vascular structures needed to simulate transient ischemic events or the immune cell coculture systems required in analyses of autoimmune interactions.^39^, ^41^, ^78^ Further research is essential to enhance the complexity of cardiac organoids by applying principles of cardiac development to achieve greater maturation and functional simulation in vitro.

### 2.5 Advancements and limitations of lung organoids

The primary function of the lungs is gas exchange, involving various cell types such as basal cells, club cells, goblet cells, and alveolar epithelial cells (AECs). AEC type I (AEC1) cells are primarily responsible for gas exchange, whereas AEC type II (AEC2) cells secrete surfactants.^79^ Specific regions from proximal airways to distal alveoli contain distinct stem and progenitor cell populations. In 2013, Barkauskas et al.^80^ revealed that AEC2 cells play a key role in alveolar maintenance and repair by forming self‐renewing “alveolospheres.” Subsequent studies have shown that the SMAD and NOTCH signaling pathways are crucial for airway stem cell proliferation and differentiation, regulating basal cell behavior.^81^, ^82^ In 2019, Sachs et al.^83^ developed human airway organoids that included basal cells, functional multiciliated cells, and mucus‐producing secretory cells. Lung organoids have become powerful tools for simulating lung physiology and disease. Lung organoids derived from human lung tumors recapitulate the morphological, histological, and genetic characteristics of the primary tumors.^84^, ^85^, ^86^ Additionally, in 2017, Chen et al.^87^ reported that the introduction of Hermansky‐Pudlak syndrome 1 mutations into lung organoids could replicate key features of fibrotic lung disease in vitro. The coronavirus disease 2019 (COVID‐19) pandemic accelerated the use of lung organoids in infectious disease research. These organoids have been utilized to study the entry mechanisms of the SARS‐CoV‐2 virus into human lungs, the pathways of viral transmission, and the mechanisms of viral shedding, revealing that AEC2 cells and the transmembrane protease serine 2 are critical for infection.^88^, ^89^, ^90^ Organoid models have also been used to study other pathogens, including explorations of the differences during infection and replication of human adenovirus types 3 and 55, assessments of potential antiviral drugs,^91^ observations of the replication adaptability of human respiratory syncytial virus and its induced innate cytokine responses in lung organoids,^92^ and analyses of avian influenza A virus tropism in human airway organoids, along with changes in cytokine and chemokine profiles.^93^

Despite these advancements, lung organoids have inherent limitations in fully replicating the cell maturity, interactions, and multicellular complexity observed in vivo. In particular, although lung organoids are primarily derived from AEC2 cells, they often lack the conditions necessary for differentiating into the AEC1 cells required for gas exchange.^94^

### 2.6 Advancements and limitations of liver organoids

The liver is a crucial hub for numerous physiological processes, including bile metabolism, vitamin metabolism, hormone regulation, blood volume control, immune system support, and endocrine regulation of growth signaling pathways.^95^, ^96^ The classic method for amplifying liver organoids was established by Huch et al.^97^ in 2013; they demonstrated that Lgr5^+^ cells from injured mouse livers could produce functional liver and bile duct organoids in vitro. In 2015, Huch et al.^98^ achieved long‐term expansion of bipotent progenitor cells from adult bile ducts in human livers, which could differentiate into functional hepatocytes. They also found that organoids derived from patients with α1‐antitrypsin deficiency and Alagille syndrome accurately reflected in vivo pathology and genetic profiles. In 2023, Zhang et al.^99^ developed 3D liver bud organoid tissues, reconstructing the structural elements of functional and vascularized organs from liver organoid tissues. Liver organoids have significantly advanced our understanding of various liver diseases; they are valuable for disease mechanism research and drug screening.^100^ In 2021, Hendriks et al.^101^ demonstrated that clustered regularly interspaced short palindromic repeats (CRISPR)‐associated protein 9 (Cas9) gene editing could be performed in organoids, facilitating studies of liver development and congenital liver diseases. In the same year, Guan et al.^102^ developed a human multilineage liver organoid model exhibiting abnormal bile ducts and fibrosis characteristics. In 2023, Zhang et al.^103^ used iPSCs to create three distinct liver organoids for the prediction of drug‐induced liver injury based on albumin production, cytochrome P450 expression, and alanine aminotransferase/aspartate aminotransferase release.

Despite rapid advancements in liver organoid research, future improvements may focus on the development of more complex coculture models that include hepatocytes, immune cells, and endothelial cells, as well as more practical and efficient microfluidic devices.^104^ Another critical area for development is the exploration of transplantation potential in liver organoids. Although Sampaziotis et al.^18^ provided evidence in 2021 that bile duct cell organoids could repair bile ducts after transplantation into human livers, additional evidence is needed to ensure the safety of such procedures for actual patient transplants.

### 2.7 Advancements and limitations of intestinal organoids

Intestinal epithelial cells are essential for the absorption and metabolism of drugs, nutrients, and water. Conventional 2D cell lines often fail to accurately replicate the complex characteristics of the gut, such as the distinct genetic profiles of epithelial cells in various regions (e.g., ileum and colon), appropriate pH levels, water absorption, and metabolic products. These limitations hinder their effectiveness in mimicking the true physiological environment within the intestine. In 2009, Clevers et al.^3^ successfully created small intestinal organoids in vitro using mouse Lgr5 intestinal stem cells for the first time. In 2021, Qu et al.^105^ developed a proliferative intestinal organoid culture system exhibiting complex crypt‐villus structures and significantly enhanced regeneration features related to tissue damage. Intestinal organoids retain the histopathological architecture of their tissue of origin. Using organoids, Lindemans et al.^106^ found that interleukin‐22 promotes epithelial regeneration mediated by intestinal stem cells. Through single‐cell RNA sequencing of cancer organoids and colorectal cancer (CRC) patient biopsies, Dmitrieva‐Posocco et al.^103^ revealed that the ketone body β‐hydroxybutyrate acts through the Hcar2‐Hopx axis, inhibiting cell viability. Fujii et al.^107^ constructed 55 CRC organoids to analyze gene mutation profiles and cellular heterogeneity, showing that the dependency on microenvironmental factors in CRC organoids is highly variable. Organoids not dependent on WNT3A/R‐spondin1 often carried mutations in the WNT signaling pathway, including adenomatous polyposis coli, catenin beta 1, and transcription factor 7 like 2.

Intestinal organoids have proven to be valuable tools for studying microbe–epithelium interactions. Since 2015, pathogens such as *Salmonella* Typhimurium*, Shigella flexneri*, and astroviruses have been shown to infect intestinal organoids.^108^, ^109^, ^110^ These findings have significantly advanced our understanding of the lifecycle of viruses, bacteria, and eukaryotic parasites in the gut, including their processes of adhesion, invasion, infection, and replication. These studies have also provided insights into the cell‐specific tropism of these pathogens for intestinal epithelial cells, goblet cells, enteroendocrine cells, and Paneth cells. In 2019, it was found that *Cryptosporidium parvum* could infect human intestinal and airway organoids, allowing new oocysts to be isolated for downstream analysis.^111^ Additionally, in 2023, researchers demonstrated that SARS‐CoV‐2 could infect intestinal organoids; its infection efficiency decreases when the farnesoid X receptor was blocked.^112^

Although intestinal organoid development has made considerable advances, several critical issues must be addressed. First, the 3D spherical structure and extracellular matrix gel used in these models limit drug and microbial access to the lumen, thereby affecting accurate assessment of intestinal permeability and drug absorption.^113^ Puschhof et al.^114^ suggested that bacterial microinjection could be a feasible solution to overcome this barrier. Second, the intestinal epithelium's sensitivity to pH, shear forces from fluid flow, and oxygen gradients poses challenges.^115^ Although microfluidic technology has been developed to address these factors, the complexity of this technology indicates that its true clinical translation remains a distant goal. Finally, the creation of multicellular organoid systems that replicate the full range of intestinal functions remains a significant challenge.

### 2.8 Challenges and innovations in organoid engineering

The current challenges facing organoids can be summarized as follows. First, most organoids lack vascularization and neural system integration. Second, the determination of cell fate and tissue organization, driven by biochemical factors and the extracellular environment, is impacted by variability in the quality control of Matrigel and cytokines, affecting the standardization and reproducibility of organoids. Finally, the achievement of high‐throughput, homogeneous, and standardized production, along with automated operations and intelligent monitoring, assessment, and control of organoids, requires the development of more advanced algorithms and methods.

**FIGURE 2:**
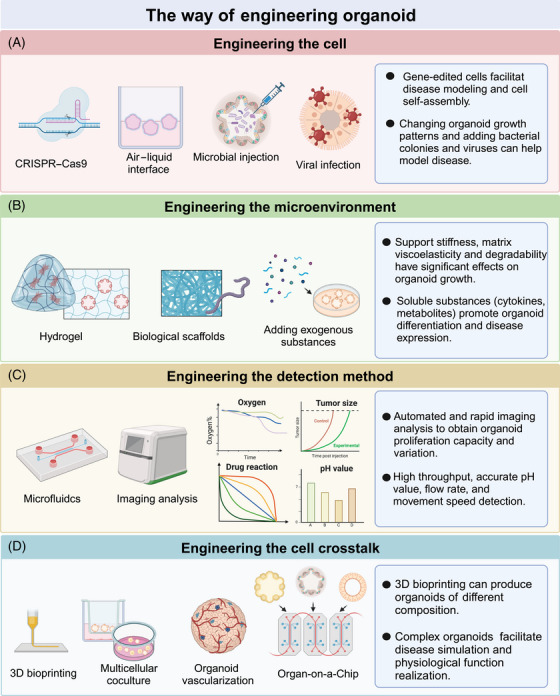
The way of engineering organoid. Engineering approaches can be applied to organoids at multiple levels. (A) Engineering the cell includes CRPISPR–Cas9, air–liquid interface, microbial injection, viral infection, and so on. (B) Engineering the microenvironment includes hydrogel, biological scaffolds, adding exogenous substances, and so on. (C) Engineering the detection method includes microfluidics, imaging analysis, and so on. (D) Engineering the cell crosstalk includes 3D bioprinting, multicellular coculture, organoid vascularization, organ‐on‐a‐chip, and so on.

At present, the engineering of organoids, which leverages bioengineering techniques, biomaterials, and AI technology, is collectively advancing the creation of predictive models, accurate disease simulations, and personalized medical strategies.^13^ Methods for engineering organoids can be broadly categorized into four areas: engineered cells, engineered microenvironments, engineered detection methods, and engineered cell–cell interactions (Figure 2).

Engineered cells encompass techniques such as CRISPR–Cas9, air–liquid interface, microbial injection, and viral injection. CRISPR/Cas9 has been widely applied in various organoids to study organ embryonic development,^116^ tumor suppression,^117^ and oncogenic transformation.^118^ The air–liquid interface method more effectively simulates the physiological environment of organoids. For example, brain organoids cultured at the air–liquid interface show improved neuron survival and axon growth.^119^ Similarly, air–liquid interface culture allows endometrial organoids to mimic the anatomical structure, cellular composition, hormone‐induced menstrual cycle changes, gene expression profiles, and dynamic ciliogenesis of the endometrium.^120^ Finally, microinjection is used to study epithelial‐microbe interactions in organoid research. For instance, the exposure of human intestinal organoids to genotoxic pks^+^ *Escherichia coli* can induce CRC mutation signatures.^121^

Organoid cultures primarily rely on extracellular matrices derived from animals or tumors. However, the complex and undefined nature of extracellular matrices sourced from murine tumor stroma limits their use in large‐scale drug screening and regenerative medicine applications.^122^ To address this challenge, Rizwan et al.^123^ designed a well‐defined viscoelastic hyaluronic acid hydrogel for organoid culture. They discovered that by mimicking the stress relaxation rate of liver tissue, they could induce the growth of cholangiocyte organoids. Additionally, bio‐scaffolds offer another approach to the modification of physiological environments within organoids. For example, whereas intestinal organoids cultured in Matrigel typically form randomly developed closed cystic structures, scaffolding can guide the formation of “mini‐guts” that more closely resemble physiological states.^124^ Brain organoids also benefit from improved support structures; Ritzau‐Reid et al.^125^ developed a microfibril scaffold to guide cavity formation in stem cells and introduced a high‐throughput method for the generation, culture, and analysis of engineered brain organoids. Furthermore, the addition of exogenous substances can simulate organoid environments or test developmental processes. For instance, the introduction of oleic acid and free fatty acids to liver organoids can mimic the formation of metabolic‐associated fatty liver disease (MAFLD), whereas the addition of troglitazone can simulate drug‐induced cholestasis.^126^ The effects of 4‐aminopyridine on iPSC‐derived brain organoids can also be evaluated in this context.^127^

Despite considerable success in the cultivation of physiologically relevant organoids, the integration of AI, microfluidics, and imaging technologies has accelerated rapid screening, cost‐effective extraction of multiscale image features, streamlined multiomics data analysis, and precise preclinical assessment and application. Microfluidic chips have been instrumental in the simulation of high‐fidelity disease models and the provision of diagnostic and therapeutic solutions. For example, Abdulla and Quintard^128^, ^129^ developed a 3D microfluidic brain organoid platform featuring dynamic fluid perturbations and integrated functional vascularized organoid chips, thereby enhancing organoid growth, maturation, and function. Additionally, AI and imaging technologies have undergone rapid advancement. For instance, Kong et al.^12^ utilized machine learning to predict drug responses in colorectal and bladder organoids. Renner et al.^130^ combined automated organoid workflows with AI‐based analysis, enabling large‐scale drug screening for Parkinson's disease and other neurodegenerative diseases. Furthermore, Singaraju et al.^131^ developed Organalysis, multifunctional image preprocessing and analysis software for cardiac organoid research, capable of calculating features such as organoid size and fluorescence intensity.

The engineering of cell–cell interactions can effectively address the challenges associated with the construction of organoids of high complexity, size, and structure. Coculture systems provide a relatively straightforward method for the enhancement of organoid complexity and realism. Examples include organoid–fibroblast coculture systems,^132^ intestinal organoid–macrophage systems,^133^ and liver cancer organoid–endothelial cell systems.^134^ Bioprinting offers a rapid and high‐throughput approach to the production of kidney organoids with consistent cell numbers and viability, enabling the assessment of the relative toxicities of drugs such as aminoglycosides.^65^ A key advantage of 3D bioprinting is its ability to more accurately replicate the tumor microenvironment by allowing precise control over multiple biomaterials, cells, and extracellular matrices within predefined architectures.^135^ Considering that drug metabolism and distribution often involve multiple organs, Nguyen et al.^136^ developed a multiorgan chip model incorporating human kidney and liver organoids to study the therapeutic effects and distribution of extracellular vesicles.

## 3 FOUR APPLICATIONS OF ORGANOIDS

**FIGURE 3:**
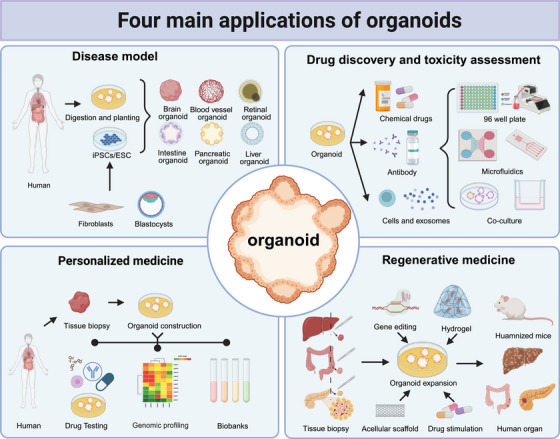
Four main applications of organoids. The main applications of organoids include the construction of disease models, drug screening and toxicity assessment, precision medicine, and regenerative medicine.

The advent of organoid technology has ushered in a new era in biomedical science. Organoids offer greater clinical fidelity than conventional models and are both cost effective and efficient, enabling more accurate disease simulation and rapid functional testing of drugs. The integration of organoids with synthetic biology, materials science, AI, and gene editing has further accelerated progress in these fields. Currently, organoids are primarily utilized in four key areas: disease modeling, high‐throughput drug screening and toxicity assessment, precision medicine, and regenerative medicine (Figure 3).

### 3.1 Organoids for disease models

The development of organoids has provided a clearer perspective on human biology, offering significant advantages over conventional disease models (Table 1). Historically, animal models have been the primary tools for studies of disease mechanisms. However, these models face challenges, including genetic and structural differences between species, ethical concerns, long cultivation periods, and high financial costs.^137^ Although cost effective and readily available, cell lines often display altered genetic backgrounds and limited physiological functionality. Additionally, cell line contamination, as encountered with HeLa cells,^138^ LO2 cells,^139^ and the MGc80‐3 cell line,^140^ has been a serious problem for biomedical research.

**TABLE 1:** Advantages and disadvantages of organoids compared with other models.

Model	Applicability	Advantages	Limitations	References
Immortalized cell lines	Disease model Drug discovery and toxicity assessment	Low cost Fewer ethical issues High‐throughput screening	Genes have been modified Lack of physiological complexity	^141^, ^142^
Primary cells	Disease model Regenerative medicine	Genomic similarity Low cost Fewer ethical issues	Issues with passage and amplification Lack of physiological complexity	^143^, ^144^
Organoids	Disease model Drug discovery and toxicity assessment Personalized medicine Regenerative medicine	Genomic similarity 3D physiological relevance Fewer ethical issues High‐throughput screening	Lack of standards Lack of complex structures	^145^, ^146^
Bioengineered organoids	Disease model Drug discovery and toxicity assessment Personalized medicine Regenerative medicine	Genomic similarity 3D physiological relevance Convenient examination Complex structure Few ethical issues	Complex operation Lack of standards	^29^, ^65^, ^147^, ^148^
Zebrafish	Disease model Drug discovery and toxicity assessment	Genomic similarity Low cost	Short lifespan Smaller tissue organs	^149^, ^150^
Mice/Rat	Disease model Drug discovery and toxicity assessment	Physiological and anatomical similarity Clearly defined genetic background	Ethical issues High cost Low‐throughput screening	^151^, ^152^, ^153^
Pig	Disease model Drug discovery and toxicity assessment Regenerative medicine	Physiological and anatomical similarity Ideal model for organ transplantation	High cost Ethical issues Low‐throughput screening Long lifespan	^154^, ^155^
Rhesus monkey	Disease model Drug discovery and toxicity assessment	Genomic similarity Physiological and anatomical similarity Behavioral and cognitive research	High cost Ethical issues Low‐throughput screening Long lifespan	^156^, ^157^

Patient‐derived organoids retain their genetic complexity and functional differences, providing high‐fidelity models for studies of organ development, tissue maintenance, and pathogenesis. As shown in Table 2, organoid models have significantly advanced research into various diseases, including genetic disorders, infectious diseases, metabolic conditions, and cancer.

**TABLE 2:** Application of organoids in modeling various diseases.

Organ	Disease	Organoid source	Main characteristic	References
Brain	Primary microcephaly	Human iPSCs cerebral organoids	Brain organoids exhibit reduced growth, defective NPC proliferation, and disrupted NPC polarity within the ventricular zone	^158^
	Cerebral cavernous malformations	Human iPSCs cerebral organoids	In brain and vascular organoids from these CCM patients, we observed enlarged clusters of endothelial channels arranged back to back and the presence of brain‐specific ECs with specialized tight junctions.	^159^
	Parkinson's disease	Human iPSCs cerebral organoids	DNAJC6 mutations cause key pathological features of PD, namely, midbrain‐type dopamine neuron degeneration, pathological α‐synuclein aggregation, increased intrinsic neuronal firing frequency, and mitochondrial and lysosomal dysfunction in human midbrain‐like organoids.	^160^
	Alzheimer's disease	Human iPSCs cerebral organoids	APOE4 exacerbates increased levels of Aβ, phosphorylated tau, synapse loss, neurodegeneration in Alzheimer's disease patient cerebral organoids.	^161^, ^162^
	SARS‐CoV‐2 infection	Human ESCs cerebral organoids	SARS‐CoV‐2 infection induces fusion between neurons and between neurons and glial cells in mouse and human brain organoids.	^43^, ^163^
	Glioblastoma	Human ASCs glioblastoma organoids	Patient‐derived glioblastoma organoids recapitulate the pathological features, cellular diversity, and genetic signatures of their corresponding parental tumors.	^164^
Retina	Retinoblastoma	Human iPSCs retinal organoids	Human retinal organoids recapitulate the molecular, cellular, and genomic features of human retinoblastoma.	^165^
	Stargardt disease	Human iPSCs retinal organoids	This retina model harbor all known major retinal subtypes such as ganglion cells, bipolar cells, horizontal cells, amacrine cells, Müller glia, and photoreceptors and successfully recapitulating the precisely orchestrated interaction between photoreceptors and retinal pigment epithelium in vitro.	^55^
	Dominant CRX‐Leber Congenital Amaurosiss	Human iPSCs retinal organoids	The CRX–LCA retinal organoid model derived from patient‐derived iPSCs showed perturbations in the photoreceptor molecular phenotype, including reduced expression of visual opsins, consistent with the loss of light responses observed clinically.	^166^
Nasal cavity	SARS‐CoV‐2 infection	Human ASCs nasal organoids	Nasal organoids exhibited a significant upregulation of cell type markers for basal cell (P63, CK5) and ciliated cell (FOXJ1, SNTN), as well as ACE2, the SARS‐CoV‐2 cellular receptor SARS‐CoV‐2 replication and higher replicative fitness of emerging variants in nasal organoids.	^167^
Thyroid	Hashimoto's thyroiditis	Human ASCs thyroid organoids	Thyroid cells in Hashimoto's thyroiditis organoids maintain properties similar to those of thyroid tissue. The expression of DTWMK gradually decreased in HT organoids, suggesting that DTYMK may be related to the progression of HT to PTC. In addition, the chemokines CCL2 and CCL3 were significantly highly expressed in HT organoids.	^168^
	Graves’ hyperthyroidism	Human ASCs thyroid follicular organoids	Human thyroid follicular organoids have intact hormone production machinery. Both mouse and human thyroid organoids express typical thyroid markers PAX8 and NKX2.1, while the thyroid hormone precursor thyroglobulin is expressed at levels comparable to tissue.	^169^
	Papillary thyroid cancer	Human ASCs papillary thyroid cancer organoids	Patient‐derived PTC organoids reflect the histology, expression profile, and genomic landscape of the parental tumor.	^170^
Heart	Myocardial infarction	Human iPSCs cardiac organoids	This study develops a human cardiac organoid disease model that recapitulates key features of the cardiac state after acute myocardial infarction at the transcriptomic, structural, and functional levels.	^171^
	Hypoplastic left heart syndrome	Human iPSCs cardiac organoids	Cardiac organoids recapitulate certain aspects of heart development.	^74^
Lung	Pseudomonas aeruginosa infection	Human ASCs lung organoid	Pseudomonas aeruginosa colonization of the apical surface of human airway epithelial organoids is promoted by periodic di‐GMP‐dependent asymmetric divisions Type 3 secretion system activity of intracellular bacteria induces goblet cell death and egress, leading to epithelial disruption, thereby increasing bacterial translocation and spread to the basolateral epithelium.	^172^
	Non‐small cell lung cancer	Human ASCs Lung organoid	NSCLC organoids also retained the histologic features and preserve the mutation and copy number landscape of their parental tumors	^85^
	SARS‐CoV‐2 infection	Human ASCs distal lung organoids	Distal lung organoids have an apical‐outward polarity with ACE2 receptors on the outside, facilitating infection of organoids with SARS‐CoV‐2 and recognizing club cells as a target population.	^173^
	Respiratory syncytial virus infection	Human ASCs airway organoids	Morphological analysis of RSV‐infected airway organoids revealed extensive epithelial abnormalities, reproducing cytoskeletal rearrangements, apical extrusion of infected cells, and syncytium formation. In addition, RSV‐infected airway organoids secreted cytokines such as IP‐10 and RANTES.	^83^
Liver	Primary sclerosing cholangitis	Human ASCs cholangiocytes organoid	PSC patient‐derived organoids show increased HLA‐DMA and CCL20 gene expression. Addition of IL17A or TNFα produces an immune response phenotype and significantly increases the secretion of proinflammatory mediators, including T cell chemokines and CCL20.	^174^
	Biliary atresia	Human ASCs cholangiocytes organoid	Biliary atresia organoids lack basal localization of the nucleus, express fewer developmental and functional markers, and display misoriented cilia. They aberrantly express F‐actin, β‐catenin, and Ezrin, have lower signals for the tight junction protein zonula occludens‐1 (ZO‐1), and display increased permeability and reduced expression of genes involved in epidermal growth factor (EGF) and fibroblast growth factor 2 (FGF2) signaling.	^175^
	Primary liver cancer	Human ASCs cholangiocytes organoid	Patient‐derived organoids retain the tissue architecture, gene expression, and genomic landscape of liver tumors and are still able to differentiate into different liver tumor subtypes.	^176^
	Liver fibrosis	Human iPSCs hepatic organoid	ARPKD mutations increase collagen abundance and production of thick collagen fibers in liver organoids, with increased activity and expansion of collagen‐producing myofibroblasts. The activities of TGFβ pathway and STAT3 signaling pathway were significantly increased.	^102^
	Hepatitis C virus infection	Human ASCs liver organoid	HCV alters the differentiation direction of organoids by inhibiting organoid proliferation and mitochondrial function while upregulating cell splicing, hepatocyte markers, and the pluripotent stem cell factor OCT4.	^177^
	Hepatitis E virus infection	Human ASCs liver organoid	Human liver organoids support the entire life cycle of HEV infection. In addition, HEV particles were observed to be primarily apically secreted. Whole‐genome transcriptome and tRNAome analyses revealed a robust host response triggered by viral replication.	^178^
	Hepatitis B virus infection	Human ASCs liver organoid	HBV‐infected organoids produce cccDNA and HBsAg, express intracellular HBV RNA and protein, and produce infectious HBV.	^179^
	Metabolic dysfunction‐related fatty liver disease	Human ESCs liver organoid	Addition of free fatty acids to liver organoids resulted in structural changes associated with nonalcoholic fatty liver disease, such as decay of the bile canalicular network and ductular reactivity.	^126^
Stomach	Helicobacter pylori (H. Pylori) infection	Human ASCs gastric cancer organoids	Gastric organoids form primitive gastric glands and caveolae‐like domains and hyperplastic zones of antral mucous cells and a variety of gastric endocrine cells. Helicobacter pylori infection results in rapid binding of the virulence factor CagA to the c‐Met receptor, activating signaling and inducing epithelial cell proliferation.	^180^, ^181^
	Gastric cancer	Human ASCs gastric cancer organoids	Gastric cancer organoids present spherical, hollow epithelial cystic structures that recapitulate the histopathological structure and genetic characteristics of the parental tumor.	^182^
	Gastroentero‐pancreatic neuroendocrine neoplasm	Human ASCs Gastroentero‐pancreatic neuroendocrine neoplasm organoids	GEP‐NEN organoids can grow independently of WNT/R‐Spondin and EGF, regardless of the presence of relevant driver gene mutations.	^183^
Intestinal	Colorectal cancer	Human ASCs colorectal cancer organoids	Organoids can reflect the “cystic and solid” tissue characteristics of epithelium. Somatic variation within the coding region of organoids is highly consistent with corresponding biopsy specimens of hypermutated and nonhypermutated patients	^184^
	SARS‐CoV‐2 infection	Human ASCs small intestinal organoids	Significant infectious virus particle titers can be detected in human small intestinal organoids readily infected with SARS‐CoV‐2, and the viral response program is strongly induced.	^185^
	Microbe infection	Human ASCs intestinal organoid	The genetic characteristics of organoids are largely determined by the interaction mechanisms between microorganisms and epithelial cells.	^114^
Pancreas	Pancreatic cancer	Human ASCs pancreatic cancer organoids	Pancreatic cancer organoids were similar to the parental tumor tissue architecture, tumor grade and differentiation pattern, and the concordance rate of molecular alterations was 91%.	^186^
	Mccune‐Albright syndrome	Human iPSCs pancreatic organoids	Mccune‐Albright syndrome form large cystic pancreatic ductal organoids and display overactive protein kinase A signaling, as evidenced by phosphorylation of target proteins including VASP.	^187^
Kidney	Kidney injury	Human iPSCs kidney organoid	Proximal tubule appeared DNA damage and kidney injury marker‐1 in multicompartment kidney organoids	^188^
	SARS‐CoV‐2 infection	Human iPSCs kidney organoid	SARS‐CoV‐2 can directly infect kidney organoids and cause cellular damage to kidney cells, subsequently leading to renal fibrosis	^189^
	Polycystic kidney disease	Human iPSCs kidney organoid	PKD organoids exhibit tubular damage and aberrant RAAS activation. In a PKD organoid xenograft model, they spontaneously form tubular cysts in mice.	^190^
	Renal cell carcinoma	Human ASCs renal cell carcinoma organoid	RCC organoids have genetic signatures that harbor RCC oncogenes, including mutations in VHL, PBRM1, PIK3CA, and BAP1, and have superior proliferation capacity compared with normal organoids.	^191^
Blood vessel	Diabetic vasculopathy	Human iPSCs blood vessel organoids	The ratio of endothelial cells to pericytes was reduced in diabetic vascular organoids, and the absolute numbers of endothelial cells and pericytes were reduced, but the localization of pericytes remained unchanged. Genes of human diabetes markers were upregulated in diabetic organoids, including angiopoietin 226, Apelin25, and TNFRSF11B26.	^25^
Blood	Hematologic malignancies	Human iPSCs bone marrow organoids	Bone marrow organoid structure recapitulated the key features of human bone marrow stroma, lumen‐forming sinusoids, and myeloid cells, including proplatelet‐forming megakaryocytes.	^192^
Salivary gland	Patient‐derived salivary gland cancer	Human ASCs salivary gland cancer organoid	Salivary gland cancer organoids recapitulated the parental tissue genotypic characterization, with >97.6% of all COSMIC annotated variants and all MYB, MYBL1, and NFIB gene rearrangements retained.	^193^
Mammary gland	Mammary gland cancer	Human ASCs human mammary organoids	Mammary organoids retain several cellular, structural, and microenvironmental features of mammary gland function.	^194^
Prostate	Advanced prostate cancer	Human ASCs prostate cancer organoid	Prostate cancer organoids retain parental genetic features, including TMPRSS2–ERG fusion, SPOP mutation, SPINK1 overexpression, and CHD1 loss.	^195^
Skin	*Staphylococcus aureus* infection	Human iPSCs skin organoids	Infection of skin organoids with *Staphylococcus aureus* resulted in cell barrier disruption and increased epidermal‐ and dermal‐derived inflammatory cytokines.	^196^
	Melanoma	Human ASCs melanoma organoids	Melanoma organoids effectively retain the pathological and genetic features of human melanoma tissue and recapitulate the highly immunosuppressive TME	^197^
	SARS‐CoV‐2 infection	Human iPSCs skin organoids	Studies have shown that KRT17^+^ hair follicle organoids can be infected by SARS‐CoV‐2, showing impaired hair follicle and epidermal development, infection of nervous system cells, and death of neurons.	^198^

Abbreviations: ASCs, adult stem cell; ACE2, angiotensin‐converting enzyme 2; AEC2, alveolar epithelial cell type II; cccDNA, covalently closed circular DNA; CCM, cerebral cavernous malformations; ECs, epithelial cells; ESCs, embryonic stem cells; EGF, epidermal growth factor; FGF2, fibroblast growth factor 2; GEP‐NEN, gastroentero‐pancreatic neuroendocrine neoplasm; HBsAg, early antigen; HEV, hepatitis E virus; iPSCs, induced pluripotent stem cell; NPC, neural progenitor cell; NSCLC, non‐small cell lung cancer; PTC, papillary thyroid cancer; PD, Parkinson's disease; PKD, polycystic kidney disease; TME, tumor microenvironment; VHL, von Hippel‐Lindau; ZO‐1, zonula occludens‐1.

#### 3.1.1 Organoids as advanced models of genetic diseases

Organoids are extensively used to model and study the mechanisms of organ‐specific genetic diseases. Organoids derived from ASCs can recapitulate the specific genetic profiles of organs and generally represent relatively mature organ development. For instance, Huch et al.^98^ reported in 2015 that liver organoids derived from human ASCs could simulate conditions such as α1‐antitrypsin deficiency and Alagille syndrome. Choi et al.^199^, ^200^ have utilized inflammatory bowel disease organoids to study the function of the metal ion transporter SLC39A8 and the butyrate‐mediated regulation of adherent‐invasive *E. coli*. iPSC‐derived organoids tend to be less mature and involve more complex induction processes. However, they are particularly suitable for studies of chromosomal variations, gene mutations, and embryonic development. For example, Li et al.^201^ developed a kidney organoid‐on‐chip model for polycystic kidney disease, discovering that cyst formation is driven by glucose transport into the lumen of the outer epithelial cells. Complex ocular diseases, such as age‐related macular degeneration and glaucoma (with >50% heritability), are often poorly replicated by animal models.^202^ Chirco et al.^203^ used retinal organoids with cone‐rod homeobox (CRX) mutations to model Leber's congenital amaurosis, whereas Huang et al.^204^ showed that retinal organoids could recapitulate key features of X‐linked juvenile retinoschisis, including retinal splitting, retinoid production defects, outer segment defects, and impaired ER–Golgi transport. Conventional cell lines and animal models also are not ideal for studies of brain development and genetic brain diseases. Since the establishment of brain organoids in 2013, researchers have used these models to study conditions such as microcephaly,^24^ tuberous sclerosis,^205^ Down syndrome,^206^ and intellectual disabilities and various cortical malformations.^207^ The advent of brain organoids has enabled researchers to investigate neurological diseases that are challenging to model in animals and to explore disease mechanisms at cellular and molecular levels within a human‐specific context.

In summary, the emergence of organoid technology has enhanced our understanding of the etiology and pathogenesis of genetic diseases, as well as the drug discovery process.

#### 3.1.2 Organoids as advanced models of infectious diseases

Organoids recapitulate numerous features of in vivo diseases while providing an excellent model for the elucidation of relationships between microorganisms—such as viruses, bacteria, and protozoan parasites—and human respiratory, digestive, and nervous systems. For instance, respiratory and distal alveolar organoids have been used to study infections by Cryptosporidium,^208^ influenza virus,^93^ and human respiratory syncytial virus,^209^ clearly demonstrating the processes of microbial infection and replication in the airways and lungs, while highlighting viral tropism and host epithelial responses. Notably, during the COVID‐19 pandemic from 2020 to 2023, organoid‐based SARS‐CoV‐2 research surged, resulting in over 200 publications that continue to have a strong impact. Lamers et al.^210^ and Salahudeen et al.^173^ found that SARS‐CoV‐2 can infect human bronchial and alveolar models; alveolar organoids expressing ACE2 receptors facilitated the infection of AEC2 or keratin 5^+^ basal cell organoids. Additionally, Han et al.^148^ identified SARS‐CoV‐2 inhibitors using lung and colon organoids, including imatinib, mycophenolic acid, and quinacrine dihydrochloride.

As the human organ most exposed to microorganisms, the gastrointestinal tract finds in organoids a valuable platform for exploring mechanisms of microbe–epithelium interactions. *Helicobacter pylori*, a well‐known gastric microorganism, was studied in gastric organoids using microinjection techniques by Bartfeld et al.^211^ in 2015. In 2022, Cao et al.^212^ used patient‐derived organoids to discover that *H. pylori* promotes gastric tumorigenesis by activating the NF‐kB‐induced RAS protein activator like 2 through the β‐catenin signaling axis. Intestinal organoids have also addressed the challenges of replicating and sustaining pathogens such as *Shigella flexneri*, typhoid, and noroviruses, overcoming limitations of conventional cell and animal models.^110^, ^213^

The development of brain organoids is crucial for studies of nervous system infections. Brain organoids have been utilized to research diseases caused by human immunodeficiency virus,^214^, ^215^ varicella‐zoster virus,^216^ SARS‐CoV‐2,^163^ Zika virus,^217^ and herpes simplex virus,^218^ revealing brain‐specific viral responses. Additionally, human brain organoids have served as models for large‐scale analyses of viral damage. Zhang et al.^219^ used organoids to elucidate the effects of influenza viruses (H1N1‐WSN and H3N2‐HKT68), severe fever with thrombocytopenia syndrome virus, and enteroviruses (EV68 and EV71) on humans.

In summary, organoids are essential for understanding the complex dynamics of pathogen infection, investigating specific mechanisms of pathogen development, and developing effective antipathogen drugs and targets.

#### 3.1.3 Organoids as advanced models of metabolic diseases

Metabolic diseases, such as obesity, diabetes, and cardiovascular diseases, impose a significant burden on modern society. The development of human‐specific adipocyte models has been challenging but essential for understanding lipid damage and glucose and lipid metabolism. Taylor et al.^220^ developed adipose organoids that incorporate immune cells to study how pro‐inflammatory microenvironments stimulate lipolysis in adipocytes under insulin resistance. Escudero et al.^221^ created vascularized and functional human beige adipose organoids, which mimic key characteristics of native beige adipose tissue, such as inducible uncoupling protein 1 expression, enhanced uncoupled mitochondrial respiration, and batokine secretion. Furthermore, organoids have been instrumental in the modeling of alcoholic liver disease and MAFLD. Wang et al.^222^ developed serum‐free liver organoids that simulate alcoholic liver disease pathophysiological changes, including oxidative stress, steatosis, inflammatory mediator release, and fibrosis, upon treatment with ethanol. In 2022, liver organoids from 24 donors were used to demonstrate genetic susceptibility to nonalcoholic steatohepatitis.^223^ Organoids have replicated MAFLD features through free fatty acid and oleic acid induction, as well as PNPLA3 mutations, resulting in lipid accumulation, enhanced inflammatory responses, and fibrosis.^223^, ^224^, ^225^ In 2023, Hendriks et al.^225^ utilized human liver organoids in CRISPR‐based target discovery and drug screening for steatosis.^225^

In summary, organoids retain specific pathogenic mutations, providing valuable insights into the underlying mechanisms of metabolic diseases and facilitating the development of personalized medicine.

#### 3.1.4 Organoids as advanced models of cancers

Due to the complexity of human tumors, clinical responses to cancer treatments can vary widely. Organoids, particularly those derived from patient‐derived ASCs, offer a way to capture this disease heterogeneity. For example, breast cancer organoids often match the histopathology, hormone receptor status, and HER2 status of the original tumors.^143^ Bladder cancer organoid models retain the genomic alterations present in parental tumors, preserving their heterogeneity.^226^ Even after long‐term culture, gastric cancer organoids maintain a genomic landscape similar to that of in vivo tumors.^227^ Non‐small cell lung cancer organoids preserve the sensitivity of the original tumors to targeted therapies and can form tumors when xenografted into nude mice.^85^ Organoids have been extensively used to screen anticancer therapies. For instance, Yan et al.^227^ demonstrated that gastric cancer organoids are sensitive to several new or investigational drugs, such as abemaciclib, nab‐paclitaxel, and the ATR inhibitor VE‐822. Pasch et al.^228^ showed that organoids can predict sensitivity to chemotherapy and radiation in various cancers, including colorectal, pancreatic, and lung adenocarcinomas. Additionally, organoids are valuable for studies of immunotherapy. They can be cocultured with immune cells, cancer‐associated fibroblasts, and other components to recreate the immune microenvironment.^229^ For example, Kong et al.^230^ cocultured tumor‐infiltrating lymphocytes with rectal cancer organoids, revealing tumor‐infiltrating lymphocyte migration into the organoids and T cell cytotoxicity. Voest et al.^231^, ^232^ developed a tumor organoid‐T cell coculture system using non‐small cell lung cancer and CRC organoids to assess T cell effector functions (including interferon γ secretion and degranulation) and their ability to kill tumor organoids. Cancer‐associated fibroblasts interact with cancer cells to enhance tumor heterogeneity, promote tumor growth, and increase resistance to chemotherapy.^233^, ^234^

Organoids are making substantial progress in cancer research and therapeutic development, addressing critical challenges such as tumor heterogeneity, immune evasion, drug resistance, and metastasis.

### 3.2 Organoids for drug discovery

Organoids have played a pivotal role in the evaluation of new drugs, significantly enhancing the efficiency and accuracy of in vitro validation and testing.^41^ Compared with primary cells and cell lines, organoids demonstrate superior organ‐specific functions and genetic characteristics. Furthermore, they are more cost effective and time efficient than animal models, such as rodents and rhesus monkeys.^235^, ^236^

Patient‐derived organoids effectively capture the heterogeneity of patient tumors and facilitate drug screening. For example, Mao et al.^237^ constructed CRC organoids to screen 335 drugs, identifying 34 with anti‐CRC activity. Fatima and Sun^238^, ^239^ also developed colorectal and liver cancer organoids to evaluate the antitumor effects of albendazole and romidepsin, respectively. Shukla et al.^240^ assessed the efficacy of 5‐fluorouracil and PRIMA‐1Met in 3D bioprinted breast cancer models, which improved organoid reproducibility. Organoids are also valuable for the evaluation of drug toxicity in organs such as the liver, kidneys, and heart. Treatments for neurological diseases often rely on neurotherapeutics, but there are significant concerns about their potential adverse effects on reproductive health. Brain chimeroids, brain chimeras, and testicular and ovarian organoids have been used to assess drug toxicity in these contexts.^241^, ^242^ For instance, Shinozawa et al.^15^ reported that iPSC‐derived liver organoids exhibited high predictive value (sensitivity: 88.7%, specificity: 88.9%) for drug‐induced liver injury. Ding^243^ utilized kidney organoids to evaluate the nephrotoxicity of aspirin, penicillin G, and cisplatin. Organoids are also proving useful for toxicity evaluation in nanomedicine research. Li et al.^244^ constructed intestinal and hepatic organoid models to assess the cytotoxicity and drug‐loaded effects of three typical metal‐organic frameworks: ZIF‐8, ZIF‐67, and MIL‐125. Additionally, considering the increasing awareness of microplastic hazards, Qin et al.^245^ used endometrial organoids to investigate microplastic pollution and its potential impact on reproductive health.

The integration of bioengineering and AI technologies with organoid technology has significantly advanced drug screening and toxicity assessment. Microfluidic technology offers a more physiologically relevant environment for organoids, enabling real‐time monitoring of factors such as oxygen tension, cytokine concentrations, and shear stress. For example, Kasendra et al.^246^ developed a duodenal small intestine chip that featured polarized cell structures, intestinal barrier function, and expression of key intestinal drug transporters. This model demonstrated improved cytochrome P450 3A4 (CYP3A4) expression and induction compared with Caco‐2 cells.^246^ Zhang et al.^247^ designed an automated microfluidic chip‐based system for continuous monitoring of organoid drug responses. Whereas single liver organoids can only reflect hepatotoxicity, multiorgan organoid models provide a more comprehensive evaluation of drug‐specific organ toxicity and metabolism. In 2017, Skardal et al.^248^ constructed a tri‐tissue organ‐on‐chip system, which included liver, heart, and lung tissues, thereby mimicking the interactive nature of the human body. In 2020, the team further advanced this concept by developing an integrated organoid chip system that incorporated liver, heart, lung, endothelium, brain, and testis organoids to study the metabolic toxicities of drugs such as 5‐fluorouracil and ifosfamide.^249^ AI technologies play a crucial role in the analysis of organoid‐based data, including microscopic images, transcriptomics, metabolomics, and proteomics, thereby facilitating high‐throughput drug screening.^250^ In 2021, Renner et al.^130^ combined automated organoid workflows with AI‐based analysis to develop next‐generation interdisciplinary high‐throughput screening methods for Parkinson's disease and other conditions. In 2022, Matthews et al.^251^ introduced a robust image analysis platform capable of automatic identification, labeling, and tracking of individual organoids in brightfield and phase‐contrast microscopy experiments, enabling the calculation of dose effects on organoid circularity, solidity, and eccentricity. In 2023, Compte et al.^252^ used pancreatic ductal adenocarcinoma organoids combined with AI‐driven live‐cell image analysis to match retrospective clinical patient responses and screen for chemotherapy drug sensitivity.

The high cost of drug development, estimated at approximately $1 billion per drug, presents significant challenges for pharmaceutical companies. These expenses contribute to elevated drug prices and can inhibit innovation, primarily due to extensive research and development efforts and the high risks associated with clinical trial failures.^253^ Major companies, including Johnson & Johnson, Roche, Takeda, and Merck, have already initiated investments in high‐throughput drug screening using organoids and organ‐on‐chip technologies.^254^, ^255^ However, before organoids can be widely adopted in clinical settings, several critical issues must be addressed by clinicians, pharmacologists, bioengineering laboratories, and regulatory agencies. First, there is a pressing need for more mature and standardized organoid models. Researchers must reach a consensus regarding the specific media, initial cell types, induction methods, and development cycles required for different organoids. Second, the integration of materials science, bioengineering, and AI technologies is crucial to improve the scalability and automation of drug screening processes. This integration will enable more accurate and high‐throughput simulations of drug metabolism across various organs, including the skin, intestine, blood vessels, liver, and kidneys. Finally, ethical and regulatory concerns surrounding the use of organoids must be thoroughly addressed. Although organoids pose relatively low ethical risks, they retain patients' genetic information, and the potential for advanced brain organoids to develop consciousness remains a significant concern.

### 3.3 Organoids for personalized medicine

Precision medicine aims to enhance disease characterization at the molecular and genomic levels, thereby improving drug screening. In recent years, extensive organoid biobanks have been established for various organs, including the brain,^164^ stomach,^227^, ^256^ liver,^257^, ^258^ kidneys,^259^ intestines^184^, ^260^ pancreas,^261^ breast,^143^ ovaries,^262^ cervix,^263^ and bladder.^264^ Organoids derived from tumors and genetic diseases can faithfully replicate the phenotypic and genomic characteristics of primary tumors. For example, Sachs et al.^143^ constructed over 100 breast cancer organoids, discovering that the DNA copy number variations and sequence changes in the organoids were consistent with those features in the original tumor tissues. Similarly, Ji et al.^257^ developed 65 human liver cancer organoids, with proteomic analysis revealing that the organoids retained the molecular and phenotypic features of the parent cancer tissues. This study demonstrated that the organoid platform could capture both intra‐ and inter‐patient heterogeneity through multiomics analyses, including histopathology, genomics, transcriptomics, and single‐cell sequencing. Moreover, organoids are valuable tools for the investigation of rare genetic diseases and identification of personalized treatments. For instance, Yao et al.^265^ and Soroka et al.^174^ created transcriptional atlases of primary sclerosing cholangitis and cholangiocyte organoids, showing that even in nontumorous conditions, organoids retained the specific pro‐inflammatory gene signatures characteristic of primary sclerosing cholangitis. Additionally, Geurts et al.^266^ constructed a biobank of intestinal organoids from 664 cystic fibrosis (CF) patients and used CRISPR gene editing to correct CF‐related gene mutations in these organoids.

Given the high‐fidelity genetic profiles of organoids, drug sensitivity testing with these models can quickly identify the most effective treatments for individual patients. For example, Yuan et al.^267^ developed organoids from gallbladder cancer, normal gallbladder tissue, and benign gallbladder adenomas. They found that the dual PI3K/HDAC inhibitor CUDC‐907 significantly inhibited the growth of various gallbladder cancer organoids while displaying minimal toxicity to normal gallbladder organoids. Similarly, Phan et al.^268^ tested 240 United States Food and Drug Administration (US FDA)‐approved or clinically developed protein kinase inhibitors on patient‐derived organoids from clear cell/high‐grade serous tumors and platinum‐resistant high‐grade serous ovarian cancer; their findings could aid clinical decision‐making. These examples underscore the potential for organoid‐based drug sensitivity testing to offer personalized treatment options and advance precision medicine.

This innovative approach aims to streamline the drug discovery process, reduce costs, and increase the success rate of new treatments by providing more accurate models of human physiology and disease. As of July 2024, a search for the keyword “organoids” on ClinicalTrials.gov identified a total of 86 registered clinical trials (Table 3). These trials are predominantly sponsored by institutions or individuals from China and the United States; most have been initiated within the past 5 years. The majority of the trials target cancers; colon cancer (15 trials), breast cancer (16 trials), ovarian cancer (five trials), and gastric cancer (eight trials) are the most common focus areas. These findings underscore the growing momentum in precision medicine research concerning patient‐derived organoids. Organoids are emerging as powerful tools for disease gene screening and personalized drug testing, which will facilitate advancements in precision medicine.

**TABLE 3:** Summary of clinical trials studies for drug testing.

Organ	Disease	Sponsor	Aiming	Status	Study Start	NCT number
Brain	Astrocytic glioma	National University Hospital, Singapore	High grade astrocytic glioma organoids serve as an ideal platform for the evaluation of drug sensitivities, accurately reflecting the patient's therapeutic response to the drugs.	Recruiting	2023‐02‐17	NCT05532397
	Recurrent high‐grade glioma	Beijing Tiantan Hospital	High grade astrocytic glioma organoids aim to evaluate the feasibility, preliminary efficacy and safety of the precision treatment strategy.	Recruiting	2022‐08‐12	NCT05473923
Head	Head and neck squamous cell carcinoma	Guy's and St Thomas' NHS Foundation Trust	The study generated organoids from patient samples and correlated the radiosensitivity and chemosensitivity of the organoids with patient survival outcomes.	Recruiting	2022‐06‐01	NCT05400239
Lung	Lung cancer	University Hospital, Geneva	This study evaluated the consistency of lung cancer organoid models in predicting the clinical efficacy of anticancer drugs.	Recruiting	2019‐04‐01	NCT03979170
	Lung cancer	Affiliated Hospital of Jiangnan University	The purpose of this study was to predict the efficacy of anticancer drugs, and to select personalized treatment regiments for patients with lung cancer.	Recruiting	2023‐02‐01	NCT05669586
	Lung cancer	Maastricht Radiation Oncology	Organoid would enable the prospective identification of “patient tailored optimal treatments.”	Completed	2017‐11‐15	NCT04859166
	Small cell lung cancer	Henan Cancer Hospital	The study first established organoids, followed by drug sensitivity testing to select appropriate clinical treatment options.	Recruiting	2024‐04‐16	NCT06406660
	Non‐small cell lung cancer	Henan Cancer Hospital	The study first established organoids, followed by drug sensitivity testing to select appropriate clinical treatment options.	Recruiting	2024‐04‐16	NCT06406608
	Non‐small cell lung cancer	K2 Oncology, Inc.	Studies assess the in vitro drug sensitivity of patient‐derived organoids and select appropriate targeted therapies or chemotherapeutic drugs.	Unknown status	2018‐01‐30	NCT03453307
	Non‐small cell lung cancer with EGFR mutation	Central Hospital, Nancy, France	This model test different molecules, osimertinib, which is a third‐generation tyrosine kinase inhibitor.	Unknown status	2021‐11‐01	NCT05136014
	Non‐small cell lung cancer	Jun Zhang, MD, PhD	Tumoroids to predict immunotherapy response in NSCLC.	Recruiting	2022‐07‐21	NCT05332925
	Non‐small cell lung cancer	Second Affiliated Hospital of Guangzhou Medical University	This Phase I study will first evaluate the safety, tolerability, and preliminary efficacy of TCR‐T cell immunotherapy in humans.	Recruiting	2018‐12‐01	NCT03778814
Esophagus	Esophageal cancer	University Medical Center Groningen	Organoids reflect patient's tumors sensitivity to therapy.	Recruiting	2017‐12‐01	NCT03283527
	Esophageal cancer	Kyungpook National University Hospital	In vitro prediction of definitive concurrent chemoradiotherapy using primary esophageal cancer organoid.	Recruiting	2015‐10‐19	NCT03081988
Liver	Intrahepatic cholangiocarcinoma	Chengjun Sui, MD	This project plans to first construct organoids. Secondly, drug screening was conducted. Then, multiomics data of organoids were used to explore the drug resistance genes.	Not yet recruiting	2023‐01	NCT05644743
	Hepatocellular carcinoma	Xiangya Hospital of Central South University	This study is aimed to establish an organoid‐on‐chips system and evaluate its efficacy in predicting the response to mfolfox6 infusion in patients with hepatocellular carcinoma.	Recruiting	2023‐03‐01	NCT05932836
	Cholangiocarcinoma	Sun Yat‐Sen Memorial Hospital of Sun Yat‐Sen University	The object of this study is to evaluate the consistency and accuracy of organoid model of cholangiocarcinoma to predict the clinical chemotherapeutic efficacy.	Recruiting	2022‐11‐30	NCT05634694
	Refractory biliary tract cancer	Mayo Clinic	To determine if drug response from a parallel ex vivo trial using patient‐derived tumor organoid correlates with clinical response to trifluridine/tipiracil plus irinotecan.	Completed	2019‐10‐18	NCT04072445
Gut	Colorectal cancer	Nanfang Hospital, Southern Medical University	The purpose of this study is to investigate whether chemotherapy guided by organoid drug test can improve the outcomes of stage IV colorectal cancer.	Recruiting	2023‐05‐01	NCT05832398
	Colorectal cancer	Peking University People's Hospital	The project will establish a screening platform for chemotherapeutic drugs and targeted drugs based on colorectal cancer organoids.	Unknown status	2021‐05‐31	NCT04996355
	Gastric or colon cancer with peritoneal carcinomatosis	Technische Universität Dresden	Chemotherapeutic agents are tested on these organoids and the organoids are analyzed with regard to genetic alterations in order to find alterations that can be addressed.	Recruiting	2022‐12‐08	NCT05652348
	Rectal cancer	Zhen Zhang	The sensitivity of irradiation and chemotherapy drugs will be tested in the organoids model.	Unknown status	2018‐08‐17	NCT03577808
	Colon cancer	D1 Medical Technology (Shanghai) Co., Ltd, China	Organoids will be exposed to the chemotherapy drugs or chemotherapy drugs combined with cetuximab used for each patient.	Unknown status	2021‐04‐15	NCT04906733
	Colorectal neoplasms	University Hospital, Akershus	A study of in vitro tailored therapy for colorectal cancer using organoids.	Recruiting	2022‐03‐28	NCT05401318
	Intestine disease	University of Erlangen‐Nürnberg Medical School	One study aimed to establish small intestinal human organoids to examine the clinical efficacy of nutritional antigens or therapeutic agents.	Recruiting	2017‐04‐01	NCT03256266
	Colorectal cancer	Funan Liu	The study aimed to evaluate the consistency of clinical efficacy and drug sensitivity results using organoids in the treatment of colorectal cancer.	Recruiting	2023‐10‐18	NCT06100016
	Advanced rectal cancer	Shanghai Minimally Invasive Surgery Center	Organoid‐based drug sensitivity and empirical neoadjuvant therapy in the treatment of advanced rectal cancer.	Not yet recruiting	2023‐01‐01	NCT05352165
	Colorectal cancer	Wuhan Union Hospital, China	The goal of this study was to use organoids to identify clinically actionable targets and predict tumor response to targeted drugs in vivo.	Not yet recruiting	2023‐07‐01	NCT05883683
	Colorectal liver metastasis	Changhai Hospital	We aim the investigate the consistency of drug sensitivity for the matched primary and metastatic tumor in patients with liver metastasis.	Recruiting	2022‐01‐01	NCT05183425
	Colorectal cancer	Chongqing University Cancer Hospital	This study used organoids for drug screening in patients with advanced/recurrent/metastatic colorectal cancer.	Recruiting	2020‐01‐01	NCT05304741
	Colorectal peritoneal metastases	Fondazione IRCCS Istituto Nazionale dei Tumori, Milano	The study used organoids derived from peritoneal metastases of colorectal cancer to select the most active drugs.	Recruiting	2021‐06‐15	NCT06057298
	Colon cancer	Institut Paoli‐Calmettes	This study used organoids to examine the efficacy of CD47‐SIRPα inhibitors on the immune microenvironment of colon cancer.	Recruiting	2023‐01‐09	NCT05955196
	Colorectal cancer	Peking Union Medical College Hospital	Validation of the 3D bioprinted tumor models as a predictive method for colorectal cancer.	Unknown status	2021‐03‐01	NCT04755907
	Rectal cancer	Helsinki University Central Hospital	Organoid guided adjuvant therapy.	Recruiting	2021‐12‐20	NCT04842006
	Colorectal cancer	Centro di Riferimento Oncologico—Aviano	This study aims to identify STARD3 is involved in colorectal cancer and to demonstrate its part in treatment sensitivity measured in tumor derived organoids.	Recruiting	2023‐05‐22	NCT06136949
Blood	Hematologic malignancy	Hematological Malignancy Organoid	This project is to compare chemosensitivity between chemotherapy combinations in bone marrow aspirates using organoid.	Recruiting	2019‐05‐16	NCT03890614
Breast	Breast cancer	Breast Cancer Organoid	Organoid model of breast cancer to predict the clinical efficacy of the drug.	Unknown status	2019‐01‐01	NCT03544047
	Breast cancer	Indiana University	Organoid model predictive of response to immunotherapies.	Recruiting	2024‐01‐22	NCT06084676
	Refractory breast cancer	Tianjin Medical University Cancer Institute and Hospital	One study first established organoid and then conducted drug sensitivity tests for the selection of clinical treatment options.	Not yet recruiting	2024‐06	NCT06438055
	Advanced breast cancer	Guangdong Provincial People's Hospital	The study provided evidence for utilizing patient‐derived organoid to personalize treatment for advanced breast cancer.	Recruiting	2024‐01‐20	NCT06102824
	Refractory breast cancer	Sun Yat‐sen University	This trial compares the efficacy and safety of organoid‐guided personalized therapy with physician‐chosen therapy for refractory breast cancer.	Recruiting	2024‐01‐15	NCT06268652
	Metastatic breast cancer	Institut Curie	A personalized tumor gram for each patient will be provided, based on the results of the drug screening.	Not yet recruiting	2024‐09‐15	NCT06459791
	Breast cancer	Second Affiliated Hospital, School of Medicine, Zhejiang University	Organoids from their tumor biopsies will be utilized to evaluate the sensitivity of chemotherapy regimen.	Recruiting	2023‐12‐06	NCT06155305
	Breast neoplasms	King's College London	The study will predict patients’ radio‐sensitivity and chemo‐sensitivity and correlation with their survival outcomes by organoid	Not yet recruiting	2024‐07‐01	NCT06468124
	Breast cancer	Xijing Hospital	Sensitivity detection and drug resistance mechanism of breast cancer therapeutic drugs based on organoid	Unknown status	2019‐01‐02	NCT03925233
	Breast cancer	National University Hospital, Singapore	This study aims to enhance patient treatment options by generating organoids derived from breast cancer patients.	Recruiting	2021‐12‐06	NCT05177432
	Breast cancer	Peking University	Development of multispecific breast cancer antibodies.	Not yet recruiting	2023‐03‐06	NCT05767931
	Metastatic breast cancer	University of Utah	Construction of organoids for genome sequencing and establishment of organoids for drug screening.	Recruiting	2021‐02‐16	NCT04450706
	Breast cancer	Institut Paoli‐Calmettes	The goal of this project is to find drugs suitable for patients with drug resistance to neoadjuvant chemotherapy.	Recruiting	2022‐11‐03	NCT04504747
	Recurrent breast cancer	University of Utah	Using organoids, the investigators will perform genomic studies and functional drug screens.	Recruiting	2023‐01‐06	NCT05464082
	Advanced breast cancer	Duke University	The purpose of this study is to assess organoid with advanced breast cancer to determine sensitivity to chemotherapy used in advanced breast cancer care.	Terminated	2022‐04‐07	NCT04655573
	Her2^+^ breast cancer	First Affiliated Hospital Xi'an Jiaotong University	This study combined genome sequencing and organoid drug sensitivity testing to establish and evaluate targeted treatment options for HER2^−^positive breast cancer.	Unknown status	2021‐01‐01	NCT05429684
Ovaries	Ovarian cancer	Sun Yat‐Sen Memorial Hospital of Sun Yat‐Sen University	Study on the consistency evaluation of organoids used in the clinical treatment of ovarian cancer with antitumor drugs.	Unknown status	2021‐11‐01	NCT05175326
	Ovarian cancer	The First Affiliated Hospital of Xiamen University	The research purpose of this study is to use organoid screen potential clinical therapeutic drugs (such as paclitaxel, gemcitabine, etc.).	Recruiting	2022‐12‐01	NCT05813509
	Ovarian cancer	Fondazione Policlinico Universitario Agostino Gemelli IRCCS	Patients derived organoids and immune cells coculture in ovarian cancer.	Recruiting	2021‐12‐20	NCT06085404
	Ovarian cancer	Chongqing University Cancer Hospital	A study testing organoids help guide precision medicine treatments for patients with ovarian cancer.	Recruiting	2022‐04‐12	NCT04768270
	Ovarian cancer	Chongqing University Cancer Hospital	Compared with the clinical efficiency of the actual drug regimen, the efficacy of the organoid drug screening model can be assessed.	Recruiting	2022‐03‐09	NCT05290961
Pancreas	Pancreatic cancer	Pancreatic Cancer Organoid	A trial of organoid drug sensitivity in neoadjuvant pancreatic cancer and prognosis	Not yet recruiting	2021‐03‐01	NCT04777604
	Pancreatic cancer	Samsung Medical Center	Development of a prediction platform for adjuvant treatment and prognosis in pancreatic cancer using analysis of organoid culture.	Recruiting	2021‐01‐31	NCT04736043
	Advanced pancreatic cancer	Changhai Hospital	This study evaluated whether organoid‐based chemotherapy guided by drug sensitivity testing could improve the treatment of advanced pancreatic cancer.	Recruiting	2021‐06‐01	NCT04931381
	Pancreatic cancer	Changhai Hospital	A trial of adjuvant chemotherapy for pancreatic cancer based on organoid drug sensitivity test.	Recruiting	2021‐06‐01	NCT04931394
	Advanced pancreatic neuroendocrine tumor	Ruijin Hospital	This study will explore the concordance between drug sensitivity test results and patients’ treatment response.	Recruiting	2024‐04‐03	NCT06246630
	Pancreatic cancer	Prof. Dr Med. Dres. H.c. Jan Schmidt, MME	The study will use organoids in clinical routine to select the best treatment for pancreatic cancer patients.	Recruiting	2022‐09‐22	NCT05351983
	Metastatic pancreatic and gastric cancer	Jianzhen Shan, MD	The aim of this study was to evaluate the consistency of in vitro tumor organoid drug sensitivity with in vivo drug treatment efficacy.	Recruiting	2023‐03‐03	NCT05842187
	Pancreatic cancer	Herlev Hospital	The study used organoids to subtype chemotherapy in the clinic and compared the responses of the organoids with those of the patients.	Active, not recruiting	2021‐07‐01	NCT05196334
	Pancreatic cancer	Ying Lv	Study uses organoids to test sensitivity of selected US FDA‐approved cancer drugs.	Unknown status	2018‐05‐01	NCT03544255
Gastric	Gastric cancer	Dong Bing Zhao	This study compared the accuracy of organoid model drug screening results with clinical drug sensitivity.	Recruiting	2023‐05‐01	NCT06196554
	Gastrointestinal tumors esophageal cancer	Jianjun Yang, MD	Study on the correlation between in vitro drug sensitivity screening and clinical efficacy of digestive tract tumor organoids	Recruiting	2022‐08‐26	NCT06332716
	Advanced gastric cancer	Shanghai Minimally Invasive Surgery Center	This study evaluated the clinical efficacy of personalized neoadjuvant therapy with organoid drug sensitivity testing compared with traditional regimens.	Unknown status	2022‐04	NCT05351398
	Gastric cancer	D1 Medical Technology (Shanghai) Co., Ltd, China	In this study, researchers tested the organoid sensitivity of chemotherapy drugs which mainly include 5‐fluorouracil, irinotecan, oxaliplatin, and paclitaxel.	Unknown status	2021‐05‐01	NCT05203549
	Early gastric cancer	Henan Cancer Hospital	Study on the potential benefits of organoid drug sensitivity screening in neoadjuvant therapy for advanced gastric cancer.	Unknown status	2022‐08	NCT05442138
	Gastric cancer	Funan Liu	This study aimed to evaluate the consistency of clinical efficacy and drug sensitivity in gastric cancer treatment using organoids.	Recruiting	2023‐10‐18	NCT06100003
	Esophagogastric carcinoma	University Hospital Heidelberg	Organoid cultures of pretreatment tumor biopsies will be established and exposed to the same chemotherapy as the corresponding patient.	Active, not recruiting	2018‐04‐15	NCT03429816
	Gastric adenocarcinoma	Xijing Hospital	This research analyzed the correlation of organoid sensitivity and the patient response.	Recruiting	2022‐07‐18	NCT05508399
Bladder	Nonmuscle invasive bladder cancer	Insel Gruppe AG, University Hospital Bern	Organoid are exposed to different drugs that are used as intravesical instillation agents in these patients (epirubicin, mitomycin, gemcitabine, docetaxel).	Recruiting	2022‐11‐17	NCT05024734
	Nonmuscle invasive bladder cancer	University of Bern	The investigators aim to use drug screens in organoid to guide neoadjuvant intravesical instillation therapy.	Not yet recruiting	2024‐10	NCT06227065
Thyroid Gland	Locally advanced thyroid cancer	West China Hospital	This research trial aims to determine the efficacy of organoid‐guided targeted therapy for patients with locally advanced thyroid cancer.	Recruiting	2021‐06‐01	NCT06482086
Bone	Osteosarcoma	Jonsson Comprehensive Cancer Center	The purpose of this study is to examine if we can predict sensitivity of osteosarcoma to different chemotherapy agents using tissue.	Recruiting	2024‐02‐12	NCT06064682
Skin	Sarcomas and melanomas	National Cancer Centre, Singapore	To perform ex vivo drug testing on patient models of sarcoma and melanoma using QPOP.	Recruiting	2020‐09‐08	NCT04986748
Multiple organs	High grade ovarian, fallopian tube, or primary peritoneal cancer	Jonsson Comprehensive Cancer Center	This study used organoid models to investigate the effects of birenapyr and carboplatin in the treatment of patients with recurrent ovarian, fallopian tube, or primary peritoneal cancer.	Withdrawn	2018‐08‐01	NCT02756130
	Colorectal cancer; pancreatic cancer; lung cancer	Weill Medical College of Cornell University	Organoids will be grown in vitro and continue to be treated with vitamin C added in culture medium to examine tumor response.	Completed	2017‐03‐29	NCT03146962
	Advanced refractory cancers	Gustave Roussy, Cancer Campus, Grand Paris	Organoids generation, culture and amplification and drug testing will be performed.	Active, not recruiting	2022‐01‐19	NCT05267912
	Refractory solid tumors	National University Hospital, Singapore	Patient derived organoids were used to select suitable chemotherapy method.	Unknown status	2019‐05‐29	NCT04279509
	Cancer	Known Medicine, Inc.	This study will assess the Known Medicine platform to predict the efficacy of cancer drugs and to validate organoid drug sensitivity.	Recruiting	2021‐01‐12	NCT05338073
	Cystic fibrosis	Kors van der Ent	Intestinal organoids can screen for drug response and identify patients with rare CFTR mutations who will respond to pipeline drugs.	Recruiting	2024‐06‐03	NCT06468527
	Cystic fibrosis	University Hospital, Montpellier	The study aims to validate the therapeutic efficacy of oligonucleotide blockers.	Recruiting	2022‐03‐30	NCT05100823

Abbreviations: CFTR, cystic fibrosis transmembrane conductance regulator; NSCLC, non‐small cell lung cancer; TCR‐T, T cell receptor chimeric T cells.

*Data source*: *ClinicalTrials.gov website* (https://clinicaltrials.gov/ct2/home).

### 3.4 Organoids for regenerative medicine

Organ transplantation remains the most definitive and effective treatment for diseases that result in irreversible organ failure. However, due to the shortage of donor organs and the adverse effects of immunosuppressive therapies, thousands of patients die each year while waiting for a transplant.^269^ The advent of organoid technology has introduced a powerful new tool to the field of regenerative medicine. Organoids have the potential to integrate, mature, vascularize, and develop specific targeted physiological functions in vivo, suggesting that in vitro cultivation of fully functional organs could one day become a reality.^270^

Significant progress has been made in the transplantation of human organoids derived from various organs, including the intestine, retina, kidney, liver, pancreas, brain, lung, and heart. Intestinal organoids have been particularly well studied. In 2014, Watson et al.^271^ generated human intestinal organoids from hESCs or iPSCs and observed significant expansion and maturation of the epithelium and mesenchyme upon transplantation into mice; the organoids exhibited functional attributes, such as permeability and peptide uptake. Xenotransplanted human ileal organoids retained their regional specificity and formed new villus structures in the mouse colon, offering a potential treatment for patients with short bowel syndrome. In July 2022, a Japanese research team conducted the first autologous transplantation using intestinal organoids cultured from the healthy gut mucosal stem cells of ulcerative colitis patients.^235^ Retinal organoids have also made substantial progress toward clinical application. Since 2011, iPSC‐derived retinal organoids transplanted into animal models of retinal diseases have shown promising results, including the development of mature and functional photoreceptors, integration with the host retina, and improvements in vision and light sensitivity.^272^, ^273^ In 2023, Hirami et al.^6^ transplanted allogeneic iPSC‐derived retinal organoid sheets into two patients with advanced retinitis pigmentosa, observing increased retinal thickness at the transplant site without severe adverse events. Organoids derived from cholangiocytes and liver cells have also undergone rapid advancement. Takebe et al.^274^ used iPSC technology to create vascularized, functional liver organoids that, when transplanted into immunodeficient mice, quickly formed functional vasculature and exhibited drug metabolism activity. In 2021, Sampaziotis et al.^18^ demonstrated that human bile duct organoids maintained their plasticity and restored function in vivo when transplanted back into the human biliary tree, despite the loss of transcriptional diversity in vitro, as shown by single‐cell RNA sequencing. In 2022, Willemse et al.^275^ enhanced the clinical translation potential of bile duct organoids by using extracellular matrix‐derived hydrogels from decellularized human or pig livers for culture, instead of the commonly used tumor‐derived basement membrane extract. Other organoids, such as those derived from the brain, pancreas, and lungs, also show regenerative potential. In 2018, Mansour et al.^39^ found that brain organoid transplantation led to integration, gradual maturation, neuronal differentiation, synapse formation, and gliogenesis, resulting in the development of a functional vascular system within the grafts. In 2020, Yoshihara et al.^276^ reported that human islet‐like organoids rapidly reestablished glucose homeostasis in diabetic immunodeficient mice. In 2019, Weiner et al.^277^ transplanted dissociated AEC2 organoids into influenza‐infected mice, demonstrating that the organoids proliferated and differentiated into AEC2 cells, which were able to support lung regeneration.

Overall, these studies suggest that organoids could become a viable alternative to conventional organ transplantation, potentially alleviating future organ shortages. Table 4 presents a selection of representative papers on human organoid transplantation from the past two decades, highlighting the progress and potential of this emerging field.

**TABLE 4:** Summary of human organoid transplantation studies.

Organ	Organoid source	Method	Model	Disease	Conclusion	Year	Country	References
Brain	iPSCs‐derived human brain organoids	After the organoids matured in vitro, they were then implanted into the cavity of the posterior cortex of the mouse brain.	NOD–SCID mice	Complex brain disorders or injuries	Human brain organoids can be implanted into the rodent brain. The transplanted organoids mature and can survive long term, develop vascularization and synaptic connections.	2018	USA	^39^
	iPSCs‐derived human brain organoids	3−5 brain organoids were implanted into the medial prefrontal cortex in both hemispheres of mouse.	NOD–SCID mice	Neurological disorders	Human brain organoids can be implanted into the rodent brain and organoids extended long projections through different brain regions.	2021	China	^286^
	iPSCs‐derived human cortical organoids	Organoids were transplanted into the primary somatosensory cortex of rats.	Athymic newborn rats	Human neural development and disease	Human cortical organoids integrate both anatomically and functionally into the rodent brain.	2022	USA	^287^
	iPSC‐derived human forebrain cortical organoids	Organoids were implanted into the visual cortex of rats in the aspiration cavity of the cortex.	Adult Long Evans rats with cyclosporine A.	Brain disorders or injuries	Brain organoids can replace mice large cortical cavities and integrating into the brain circuitry, which demonstrates neural tissues repair of brain circuitry.	2023	USA	^17^
	iPSCs‐derived human brain organoid	Human brain organoids were transplanted onto the pial vessels of the hippocampal tail in mice by craniotomy.	Immune deficient NOD.Cg‐Prkdc^scid^/J mice	Autism with macrocephaly	Brain organoids with human microglia acquire human‐specific transcriptomic signatures, and engage in surveilling the human brain environment while reacting to perturbations.	2023	USA	^41^
Eye	ESCs‐derived human retinal organoids	Subretinal retinal organoids transplantation was performed in RCS rats (2 × 10^5^ cells in 2 µL) or in rd1 mice (1 × 10^5^ cells in 1 µL)	RCS rats and rd1 mice	Retinal degeneration diseases	C‐Kit^+^/SSEA4^−^ cells from hESC‐derived retinal organoids improved vision and preserve the retinal structure.	2019	China	^288^
	ESCs‐derived human retinal organoids	Retinal Sheet (0.7−1.3 × 0.6 mm) were delivered to the subretinal space of the left eye using a custom implantation instrument.	RCS rats	Retinal degeneration diseases	The transplantation of retinal organoids derived improves visual function in nude RCS rats through both cell replacement and cell rescue.	2020	USA	^289^
	ASCs‐derived lacrimal gland organoids	Approximately 5 µL of organoid suspension (approximately 1.5 × 10^4^ cells) was instilled into the mouse lacrimal gland using an insulin needle.	NSG mice	Tear gland pathologies	Orthotopic transplantation in mice engrafts and produces mature tear products.	2021	Netherlands	^290^
	iPSC/ESCs derived human lacrimal gland organoids	1–5 organoids cultured in Matrigel were placed in rat lacrimal gland tissue, then the external skin was sutured.	F344/NJcl‐rnu/rnu rats	Dry‐eye syndrome	When transplanted into the vicinity of the recipient rat's eye, it undergoes functional maturation, forms a cavity and produces tear film proteins.	2022	Japan	^291^
	iPSCs‐derived human retinal organoids	Three sheets would be transplanted with a single insertion of the plastic cannula, performed at sites where the inner retinal layer and retinal epithelium.	2 advanced retinitis pigmentosa	Retinitis pigmentosa	Allogeneic retinal organoid sheets survived in a stable condition for 2 years without immune rejection or unexpected overgrowth.	2023	Japan	^6^
Thyroid	ASCs‐derived human thyroid organoids	6 × 10^5^ dispersed cells were transplanted underneath the kidney capsule of hypothyroid mice.	Hypothyroid mouse model	Hypothyroidism	Thyroid gland organoids demonstrated their capability to form thyroid gland tissue beneath the kidney capsule in a hypothyroid mouse model.	2021	Netherlands	^292^
Thymus	iPSCs‐derived human thymus organoids	1−3 × 10^5^ thymus organoids were transplanted underneath the kidney capsules of mice.	NSG mice	Immune deficient disorders	Human thymic organoids can support the de novo generation of diverse functional human T cell populations after transplantation into mice.	2022	USA	^293^
Breast	iPSCs‐derived human mammary organoids	The orthotopic transplantation of human mammary organoids to the mammary gland‐cleared fat pad of mice was conducted.	NSG mice	Postmastectomy breast reconstruction	Mammary organoids to generate mammary gland‐like structures in both in vitro and in vivo conditions, laying the foundation for breast reconstruction.	2022	China	^294^
Heart	ESCs‐derived human cardiac organoids	Cardiac organoids were sutured to the internal abdominal muscle of each nude mouse by ventral middle laparotomy.	B6NU mice	Myocardial development	Ectopic implantation of cardiac organoids into the peritoneal cavity of immunodeficient mice induces neovascularization, Ca^2+^ handling, and upregulation of genes for ion channel proteins.	2019	Iran	^76^
Lung	ASCs‐derived human airway organoids	Airway organoids were transplanted, embedded within Matrigel, under the kidney capsule of mice.	NSG mice	Lung regeneration	Heterotopic transplantation of human airway organoids into mice can recruit host vascular cells and express lineage specific differentiation of epithelial cells.	2018	USA	^295^
	ESCs‐derived human lung organoids	Organoids were transplanted under the kidney capsule of the mice (2−3 drops of organoids per mouse).	Immunodeficient B‐NSG mice	Lung regeneration	Lung organoids can colonize mouse lungs, and SOX9 inactivation has limited effects on alveolar cell differentiation and lung regeneration in injured mice.	2021	China	^296^
Liver	iPSC‐derived vascularized human liver organoids	1 × 10^7^ cell‐equivalent liver buds were transplanted under the renal capsule of mice.	Alb‐Tk‐NOG mice	Liver failure	Liver organoids colonize the liver, enabling functional rescue of acute liver failure via transplantation.	2017	Japan	^297^
	iPSC‐derived human liver organoids	PGEC‐LBs were transplanted into an acute liver failure mouse model by kidney capsule transplantation.	Alb‐TRECK/SCID mice	Liver failure	Transplantation of PGEC‐derived liver bud organoids showed therapeutic potential against fulminant liver failure.	2018	Japan	^298^
	iPSC‐derived human liver organoids	1 × 10^5^/kg liver organoids were transplanted into infantile piglets without PDV ligation and 3.5 × 10^4^/kg liver organoids were transplanted into 28‐day‐old infantile piglets with PDV ligation, respectively.	Humanized pig	Liver regeneration	Transplanting liver organoids derived from human iPSCs through the portal vein is a safe procedure, as ligation of the ductus venosus effectively prevents their translocation to extrahepatic sites.	2020	Japan	^299^
	iPSCs‐derived human liver organoids	1 × 10^5^ cells in 150 µL of PBS were injected into spleen.	FRG mice with alcohol	Alcohol‐related liver disease	Liver organoids can colonize immunodeficient mice. When exposed to ethanol, organoids have an alcohol‐related liver disease phenotype.	2023	Korea	^300^
	iPSCs‐derived human liver organoids	5 × 10^6^ liver organoids were transplanted intraperitoneally using a 1 mL syringe and 22‐gauge needle.	FRG mice with acetaminophen or hepatectomy	Liver failure	Liver organoid therapy improves survival in mice with liver failure after hepatectomy and improves hyperammonemia and hypoglycemia by providing liver function.	2024	China	^301^
Bile duct	ASCs‐derived human cholangiocyte organoids	PGA scaffolds populated with bile duct organoids for surgical bile duct replacement.	Extrahepatic biliary injury NSG mouse	Biliary injury	After cholangiocyte organoids are transplanted into mice, they form tissue‐like structures that retain the characteristics of the bile duct, replacing the natural common bile duct.	2017	UK	^302^
	ASCs‐derived human cholangiocyte organoids	Cholangiocytes organoids were infused through the cannula in the gallbladder in a total volume of 1 µL/g of total body weight, at a maximum speed of 1 µL/s.	Human liver	Disorders of the biliary system	After transplantation, human cholangiocyte organoids can colonize human liver, restore the expression of region‐specific markers.	2021	UK	^18^
Pancreas	iPSCs‐derived human pancreatic organoids	1 × 10^6^ pancreatic organoids were orthotopically transplanted into the murine pancreas.	Athymic NMRI‐nu/nu mice	Cystic fibrosis	Pancreatic organoids can form normal pancreatic duct and acinar tissue in mice, similar to the fetal human pancreas.	2017	Germany	^303^
Kidney	iPSCs‐derived human kidney organoids	Kidney organoids were bisected and transplanted under the renal capsules of kidneys mice	NOD–SCID mice	Kidney development	After transplantation, kidney organoids develop vascularization, functional glomerular perfusion.	2018	Netherlands	^304^
	iPSCs‐derived human kidney organoids	10–20 kidney organoids were subsequently implanted beneath the kidney capsule of mice by a PE50 tube.	NOD–SCID mice	Chronic kidney disease	Kidney organoids transplanted into the renal capsule of immunodeficient mice need to consider safety and their maturity needs to be improved.	2019	Korea	^305^
	iPSCs‐derived human kidney organoids	Kidney organoids transplanted onto the left kidney capsule of mice using 24 GHz catheter	NOD–SCID mice	Kidney disease	Transplanting kidney organoids can reduce the generation of off‐target cells.	2019	USA	^306^
	iPSCs‐derived human kidney organoids	10–20 kidney organoids were transplanted with 0.1% kidney dECM into the renal subcapsular space of mice with a 23‐gauge syringe needle.	NOD–SCID mice	Fabry nephropathy and vascular disease	Kidney organoids can be transplanted into mouse kidneys and recruit endothelial cells to maintain vascular integrity.	2022	Korea	^307^
Intestinal	iPSCs‐derived human intestinal organoids	Single intestinal organoid was transplanted under the kidney capsule of mice.	NSG mice	Infectious or allergen‐driven enteric disease	Intestinal organoids colonizing the kidney capsule demonstrated functional human immune cell infiltration.	2023	USA	^283^
	ESCs‐derived human intestinal organoids	Intestinal organoids were transplanted into the renal subcapsular space of mice.	NSG mice	Human intestinal development	Intestinal organoids colonize the intestine, mimicking human fetal intestinal development.	2023	USA	^308^
	iPSCs‐derived human colonic organoids	Single human colonic organoids were transplanted under the kidney capsule of mice.	NSG mice	Inflammatory colon diseases	human colonic organoids macrophages establish residency, phagocytose bacteria and respond to commensal and pathogenic bacteria in the lumen.	2023	USA	^309^
Endometrium	ASCs‐derived human endometrial organoids	Approximately 10 µL of Matrigel was infused into the uterine horns, followed by the slow delivery of organoids (1 × 10^6^ cells) into the uterine horn.	BALB/c‐nude mice	Asherman's syndrome	Endometrial organoids were successfully implanted into mice and restored the metabolic state of the endometrium in Asherman syndrome mice.	2024	Korea	^310^
Skin	iPSCs‐derived human skin organoid	Skin organoids (6 × 10^6^ hiPSC‐KCs, 3 × 10^6^ hiPSC‐FBs and 3 × 10^6^ hiPSC‐ECs) was performed by performing an 8 mm punch biopsy on the dorsal skin of mice to create full‐thickness skin wounds.	NOD.Cg‐Prkdcscid Il2rgtm1WjI/SzJ mice	Skin regeneration and wound healing	Complex skin organoids can heal deep wounds in mice, significantly accelerating angiogenesis.	2021	Austria	^311^
Vessel	iPSCs‐derived human blood vessel	Vascular organoids were transplanted under the kidney capsule of mice.	NSG mice with 40 mg/kg Streptozotocin	Diabetic vasculopathy	Blood vessel organoids exhibit morphological, functional, and molecular features of human microvasculature.	2019	Austria	^25^

Abbreviations: ASCs, adult stem cell; ALB, albumin; dECM, decellularized extracellular matrix; EC, endothelial cells; ESCs, embryonic stem cells; FB, fibroblasts; FRG, Fah^− /−^/Rag2^−/−^/ Il ‐ 2rg^−/−^; iPSCs., induced pluripotent stem cell; KC, keratinocyte; NSG, NOD/SCID/γ‐chain knockout; NOD–SCID, nonobese diabetic/severe combined immunodeficiency; NSG, NOD. Cg‐*Prkdc^scid^ IL2rg^tm1Wjl^/*SzJ, PGEC‐LBs; RCS, Royal College of Surgeons; PDV, patent ductus venosus; PGEC‐LB, posterior gut endoderm cells‐liver buds; PGA, polyglycolic acid.

Despite their promising potential in regenerative medicine, organoids face several challenges and limitations. First, organoid size and expansion are critical for their clinical application. Transplantable organoids must be as large and numerous as possible, but their proliferation rate is significantly lower than that of cancer cell lines. One feasible approach to address this challenge involves the use of rotating bioreactors. For instance, Qian et al.^217^, ^278^ developed a small rotating bioreactor, known as SpinΩ, to optimize brain organoid maturation while reducing costs. Second, organoid maturation presents a significant hurdle. The culture requirements for organoids differ substantially from those for conventional monolayer cell cultures. Most iPSC‐ and hESC‐derived organoids tend to resemble fetal rather than adult cells, which makes it difficult to replicate complete vascularization and neural networks in vitro.^279^, ^280^ Although scientists have developed various bioreactors to meet the oxygenation, lineage progression, and 3D spatial organization needs of organoids, progress remains challenging.^281^ Limitations such as high fluid shear and impeller instability can negatively affect shear‐sensitive organoid cultures. Although technologies such as SpinΩ have significantly advanced the bioexpression of forebrain organoids, 3D printing technology remains exceedingly complex for widespread use.^282^ Third, the need for robust preclinical validation models poses another challenge. The immune‐deficient models essential for preclinical evaluation are costly, limiting their accessibility and use.^283^, ^284^ Finally, the assurance of transplantation safety is a central requirement. Before organoid transplantation can become a standard practice, concerns regarding potential tumorigenicity and the differentiation capacity of organoids must be addressed.^66^ The establishment of protocols that comply with Good Manufacturing Practice guidelines to evaluate the long‐term risks of in vitro‐cultured organoids and their potential tumorigenicity post‐transplantation offers a rational solution to these safety concerns.^285^

## 4 CONCLUSIONS AND PERSPECTIVE

### 4.1 Future organoid research

Organoids have made substantial progress in disease simulation, drug development, patient precision medicine, and regenerative medicine. A promising future direction for organoid research lies in the development of multiorganoid systems that use microfluidics and biomaterials to integrate organ systems, thereby enabling highly detailed disease modeling. Perfusable microfluidic devices can ensure the efficient delivery of nutrients, gradient pressure, and oxygen to organoids, which are crucial for the maintenance of their viability and functionality. By manipulating biomaterial parameters such as cell‐binding ligands, structural geometry, and mechanical properties, researchers can control organoid development and disease progression.^312^, ^313^ Because diseases, drugs, and microorganisms impact organ function and structure, as well as the organ's microenvironment, “multiorganoid chips” are poised to become a cutting‐edge technology for the evaluation of new drug efficacy and toxicity.^314^

Moreover, the integration of organoid technology, tissue engineering, and AI offers exciting possibilities for enhanced understanding of organoid engineering principles.^13^ Studies have begun to explore the use of AI in hydrogel design, optimization, and its applications in biomedicine.^315^ For instance, Kowalczewski et al.^316^ utilized unsupervised machine learning to cluster and refine cardiac organoids based on functional similarity, revealing unique features related to their geometric design. Additionally, Lefferts et al.^317^ introduced OrgaSegment, a deep learning segmentation model based on MASK‐RCNN, which quantifies organoid swelling. This model can differentiate among organoids with various CF transmembrane conductance regulator (*CFTR*) mutations and assess their responses to *CFTR*‐modulating drugs. The integration of organoid technology with AI has enabled a more comprehensive analysis of organoid behaviors, intricate cellular interactions, and dynamic responses to various stimuli.^318^ AI is revolutionizing our approach to in vitro modeling, pushing the boundaries of biomedical research.

### 4.2 Challenges and limitations of organoid research

Although the field of organoids holds broad prospects for various applications, it faces several significant challenges and limitations. First, organoid technology bridges the gap between cell lines and in vivo models; however, most organoids lack the surrounding stromal cells, immune cells, and vascular endothelial cells necessary for comprehensive modeling.^319^ For example, liver organoids often lack hepatocyte zonation and essential components of metabolic fatty liver disease pathogenesis, such as vasculature, immune cells, and neural innervation.^320^ Second, the organoid industry is hindered by a lack of standardization, a situation exacerbated by the rapid progress in engineered organoids. Factors affecting reproducibility include batch‐to‐batch variation (e.g., patient tissue heterogeneity, as well as timing and method of iPSC induction), culture conditions (e.g., cytokine concentration, matrix gel concentration, and matrix gel composition), and cell composition and organoid structure.^321^ The resolution of these issues will require collaborative efforts from biomedical scientists, clinicians, and regulatory agencies to standardize and homogenize organoid technology, which would facilitate the transition from scientific research to clinical practice and enable the production of larger‐scale organoids for drug screening.^235^ Third, the cultivation of human organoids often relies on mouse‐derived extracellular matrix substitutes, such as Matrigel and basement membrane extracts. However, issues such as the tumor origins of these materials, batch‐to‐batch variability, high costs, and safety concerns impact the reproducibility and practicality of experimental results. Additionally, the presence of unknown pathogens and potential immune responses make these substrates unsuitable for clinical transplantation.^322^ Recent advances have introduced alternative materials, such as graphene oxide, biomimetic hydrogels, and self‐assembling peptide hydrogels, which offer promising replacements for Matrigel and enhance the development of advanced organoid models.^323^, ^324^, ^325^ Although organoids generally raise fewer ethical issues than other technologies, there are ongoing ethical deliberations concerning potentially controversial entities such as brain organoids and embryoid bodies.^326^ Much of this debate centers on the ethical status of such organoid models and the implications of their use.^327^

In summary, although the field of organoids is rapidly advancing, several challenges remain, including issues with standardization, matrix gel contamination, bioethics, and complexity. The resolution of these challenges through the construction of engineered organoids, the development of new materials, and the establishment of industry standards will be crucial for future progress in this field.

### 4.3 Conclusion

In summary, organoids have demonstrated immense potential in disease modeling, drug development, and precision medicine. They are capable of replicating and exhibiting complex biological processes, including multicellular and organ interactions, tumor immune microenvironments, and host‐pathogen interactions. Moreover, the integration of organoids with advanced materials science, AI, and gene editing has reshaped our understanding of diseases and translational therapies, significantly advancing our efforts to address and conquer human diseases.

## AUTHOR CONTRIBUTIONS

*Conceptualization; data curation; formal analysis; writing—original draft*: Qigu Yao. *Data curation; formal analysis*: Sheng Cheng. *Data curation; formal analysis*: Qiaoling Pan. *Investigation*: Jiong Yu. *Investigation*: Guoqiang Cao. *Conceptualization*: Lanjuan Li. *Conceptualization; funding acquisition; writing—review and editing*: Hongcui Cao. All authors have read and approved the final version of the manuscript.

## CONFLICT OF INTEREST STATEMENT

The authors declare no conflict of interest.

## ETHICS STATEMENT

Not applicable.

## Data Availability

The data that support the findings of this study are available from the corresponding author upon reasonable request.
